# Long Covid in adults discharged from UK hospitals after Covid-19: A prospective, multicentre cohort study using the ISARIC WHO Clinical Characterisation Protocol^^^^^^^

**DOI:** 10.1016/j.lanepe.2021.100186

**Published:** 2021-08-06

**Authors:** Louise Sigfrid, Thomas M. Drake, Ellen Pauley, Edwin C. Jesudason, Piero Olliaro, Wei Shen Lim, Annelies Gillesen, Colin Berry, David J. Lowe, Joanne McPeake, Nazir Lone, Daniel Munblit, Muge Cevik, Anna Casey, Peter Bannister, Clark D. Russell, Lynsey Goodwin, Antonia Ho, Lance Turtle, Margaret E. O'Hara, Claire Hastie, Chloe Donohue, Rebecca G. Spencer, Cara Donegan, Alison Gummery, Janet Harrison, Hayley E. Hardwick, Claire E. Hastie, Gail Carson, Laura Merson, J. Kenneth Baillie, Peter Openshaw, Ewen M. Harrison, Annemarie B. Docherty, Malcolm G. Semple, Janet T. Scott, ISARIC4C investigators

**Affiliations:** a Clinical Research Fellow, Public Health Specialist, ISARIC Global Support Centre, Centre for Tropical Medicine and Global Health, University of Oxford, Oxford, UK; b Clinical Research Fellow, Centre for Medical Informatics, University of Edinburgh, Edinburgh, UK; c Medical Student, University of Edinburgh Medical School, Edinburgh, UK; d Consultant in Rehabilitation Medicine, NHS Lothian, Edinburgh, UK; e Professor of Poverty Related Infectious Diseases, ISARIC Global Support Centre, Centre for Tropical Medicine and Global Health, University of Oxford, Oxford, UK; f Honorary Professor of Respiratory Medicine, Department of Respiratory Medicine, Nottingham University Hospitals NHS Trust and University of Nottingham, UK; g Senior Clinical Trial Nurse, ISARIC Global Support Centre, Centre for Tropical Medicine and Global Health, University of Oxford, Oxford, UK; h Professor of Cardiology and Imaging, Institute of Cardiovascular and Medical Sciences, University of Glasgow; i Consultant in Emergency Medicine, NHS Greater Glasgow and Clyde, Emergency Department, Glasgow, UK; j Nurse Consultant in Clinical Research, School of Medicine, Dentistry and Nursing, University of Glasgow, UK; k Senior Clinical Lecturer in Critical Care, Usher Institute, University of Edinburgh, Edinburgh, UK; l Professor of Paediatrics, Department of Paediatrics and Paediatric Infectious Diseases, Institute of Child's Health, Sechenov First Moscow State Medical University (Sechenov University), Moscow, Russia; m Clinical Senior Lecturer, Inflammation, Repair and Development Section, National Heart and Lung Institute, Faculty of Medicine, Imperial College London, London, UK; n Medical Student, Brighton and Sussex Medical School, Brighton, UK; o Clinical Lecturer in Infectious Diseases, Centre for Inflammation Research, University of Edinburgh, UK; p Specialist Registrar in Infectious Diseases and General Internal Medicine, Tropical and Infectious Diseases Unit, North Manchester General Hospital, Delaunays Rd, Manchester, UK; q Clinical Research Fellow, National Institute of Health Research (NIHR) Health Protection Research Unit in emerging and zoonotic infections, Institute of Infection, Veterinary and Ecological Sciences, University of Liverpool, Liverpool, UK.; r Clinical Senior Lecturer/Consultant in Infectious Diseases, MRC-University of Glasgow Centre for Virus Research, Glasgow, UK; s Senior Clinical Lecturer, NIHR Health Protection Research Unit in emerging and zoonotic infections, Institute of Infection, Veterinary and Ecological Sciences, University of Liverpool, Liverpool, UK; t Consultant in Infectious Disease, Tropical and Infectious Disease Unit, Liverpool University Hospitals NHS Foundation Trust, Liverpool, UK; u Patient-advocate, Long COVID Support, Birmingham, UK; v Trials Coordinator, Liverpool Clinical Trials Centre, University of Liverpool, Liverpool, UK; w Project Administrator, Institute of Infection, Veterinary and Ecological Sciences (IVES), University of Liverpool, Liverpool, UK; x Project Administrator, Institute of Infection, Veterinary and Ecological Sciences (IVES), University of Liverpool, Liverpool, UK; y Data Base Developer, National Institute of Health Research (NIHR) Health Protection research Unit in Emerging and Zoonotic Infections, Liverpool, UK; z Institute of Infection and Global Health, Faculty of Health and Life Sciences, University of Liverpool, Liverpool, UK; aa Project Manager, National Institute of Health Research (NIHR) Health Protection research Unit in Emerging and Zoonotic Infections, Liverpool, UK; ab Lecturer in Public Health, Institute of Health and Wellbeing, University of Glasgow, Glasgow, UK; ac Head, ISARIC Global Support Centre, Centre for Tropical Medicine and Global Health, University of Oxford, Oxford, UK; ad Head of Data, ISARIC Global Support Centre, Centre for Tropical Medicine and Global Health, University of Oxford, Oxford, UK; ae Senior Clinical Research Fellow, Roslin Institute, University of Edinburgh, Edinburgh, UK; af Prof. of Experimental Medicine, National Heart and Lung Institute, Imperial College, London UK; ag Director Centre for Medical Informatics, Usher Institute, University of Edinburgh, Edinburgh, UK; ah Wellcome Clinical Research Career Development Fellow, Centre for Medical Informatics, Usher Institute, University of Edinburgh, Edinburgh, UK; ai Consultant in Intesnive Care Medicine, Intensive Care Unit, Royal Infirmary Edinburgh, Edinburgh, UK; aj Professor of Child Health and Outbreak Medicine, Health Protection Research Unit In Emerging and Zoonotic Infections, Institute of Infection, Veterinary and Ecological Sciences, University of Liverpool, UK; ak Consultant in Respiratory Medicine, Alder Hey Children's NHS Foundation Trust, Eaton Road, Liverpool, UK; al Clinical Lecturer in Infectious Disease, MRC-University of Glasgow Centre for Virus Research, Glasgow, UK; am Clinical Lecturer, Division of Infection and Global Health Research, University of St Andrews

**Keywords:** Covid-19, post-acute Covid-19, long-Covid, post-Covid, sequelae, long-term outcomes, quality of life

## Abstract

**Background:**

This study sought to establish the long-term effects of Covid-19 following hospitalisation.

**Methods:**

327 hospitalised participants, with SARS-CoV-2 infection were recruited into a prospective multicentre cohort study at least 3 months post-discharge. The primary outcome was self-reported recovery at least ninety days after initial Covid-19 symptom onset. Secondary outcomes included new symptoms, disability (Washington group short scale), breathlessness (MRC Dyspnoea scale) and quality of life (EQ5D-5L).

**Findings:**

55% of participants reported not feeling fully recovered. 93% reported persistent symptoms, with fatigue the most common (83%), followed by breathlessness (54%). 47% reported an increase in MRC dyspnoea scale of at least one grade. New or worse disability was reported by 24% of participants. The EQ5D-5L summary index was significantly worse following acute illness (median difference 0.1 points on a scale of 0 to 1, IQR: -0.2 to 0.0). Females under the age of 50 years were five times less likely to report feeling recovered (adjusted OR 5.09, 95% CI 1.64 to 15.74), were more likely to have greater disability (adjusted OR 4.22, 95% CI 1.12 to 15.94), twice as likely to report worse fatigue (adjusted OR 2.06, 95% CI 0.81 to 3.31) and seven times more likely to become more breathless (adjusted OR 7.15, 95% CI 2.24 to 22.83) than men of the same age.

**Interpretation:**

Survivors of Covid-19 experienced long-term symptoms, new disability, increased breathlessness, and reduced quality of life. These findings were present in young, previously healthy working age adults, and were most common in younger females.

**Funding:**

National Institute for Health Research, UK Medical Research Council, Wellcome Trust, Department for International Development and the Bill and Melinda Gates Foundation.

**Research In Context**

**Evidence before this study**

• Long-term symptoms after hospitalisation for Covid-19 have been reported, but it is not clear what impact this has on quality of life.
• It is not known which patient groups are most likely to have long-term persistent symptoms following hospitalisation for Covid-19, or if this differs by disease severity.

**Added value of this study**

• More than half of patients reported not being fully recovered several months after onset of Covid-19 symptoms.
• New or worse disability was reported in a quarter of participants.
• EQ5D-5L summary index suggests that quality of life was significantly reduced by about 10%.
• Females under 50 and those with more severe acute disease in-hospital had the worst long-term outcomes.

**Implications of all available evidence**

 

• Policy makers need to ensure there is long-term support for people experiencing long-Covid and should plan for lasting long-term population morbidity. Funding for research to understand mechanisms underlying long-Covid and identify potential interventions for testing in randomised trials is urgently required.

Alt-text: Unlabelled box

## 1 Introduction

Our understanding of long-term outcomes after acute Covid-19 remains limited. It is becoming increasingly evident that some patients who have had acute Covid-19 go on to experience persistent symptoms, known as long-Covid or post-Covid syndrome [1]. Several studies in hospitalised and community settings have identified that those with Covid-19 frequently develop long-term symptoms and a range of sequelae affecting the kidneys, lungs and heart [1], [2], [3], [4], [5]. These symptoms appear to overlap with other post-viral syndromes and with the challenges faced by patients recovering from other critical illness with post-intensive care syndrome (PICS), such as muscle weakness, fatigue, and sleep disturbance [6], [7], [8], [9], [10]. Yet, understanding the impact Covid-19 has on patient reported outcome measures, including quality of life, has not yet been fully characterised [11].

Many clinical trials or studies that aim to characterise the immediate course of Covid-19 have used mortality as a primary outcome [12,13]. This has demonstrated that patients in older age groups and those who have pre-existing comorbidities are at higher risk of dying from the disease [14], [15], [16], [17]. Nonetheless, most people with Covid-19 will survive the initial acute infection and data on what happens to these individuals in the long-term are lacking. The large number of people affected by Covid-19 and the growing evidence of long-term sequelae highlights the importance for policy makers, society and healthcare systems to understand the difficulties faced by those suffering from long-Covid [4,18,19]. Understanding the burden of disease, and who is at greatest risk of developing long-term complications, may help to target preventative strategies and provide effective support for affected individuals to improve Covid-19 outcomes and reduce risk of widening health inequalities by inadequate rehabilitation and recovery support. Identifying which patient groups are most likely to be affected could provide data to guide policy and aid future research to identify disease mechanisms, and to formulate and test new interventions.

The objective of this study was to characterise long-term patient reported outcomes in individuals who survived hospitalisation for Covid-19, in those who engaged with post hospital follow-up, using the International Severe Acute Respiratory and emerging Infections Consortium (ISARIC) WHO Clinical Characterisation Protocol (CCP-UK) and follow-up protocol [20].

## 2 Methods

### 2.1 Study design and setting

The ISARIC WHO Clinical Characterisation Protocol (CCP) was first developed by international consensus in 2012 to respond to any emerging or re-emerging pathogen of public health interest [21]. It was activated in the UK in response to the SARS-CoV-2 pandemic on 17th January 2020. Study information including the CCP-UK and post hospital follow-up protocol, standardised case report forms, study information and consent forms, are available on the ISARIC4C.net website. Hospitals providing acute care throughout the United Kingdom were eligible to enrol participants into the study. This analysis is reported in line with the Strengthening the Reporting of Observational Studies in Epidemiology (STROBE) guidelines [22].

### 2.2 Participants

Patients aged 18 years and over, admitted to hospital between 17^th^ January to 5^th^ October 2020 with confirmed or highly suspected SARS-CoV-2 infection at 31 centres, who consented to be contacted for post hospital follow-up and were discharged at least 90 days ago were eligible for inclusion. Participants experienced post-Covid sequelae without formal treatment as management pathways for long Covid were not available at this time. Confirmation of SARS-CoV-2 was by reverse-transcriptase polymerase chain reaction (RT-PCR). Individuals with clinically diagnosed highly suspected, Covid-19 were also eligible for inclusion, given that SARS-CoV-2 was an emergent pathogen in the earlier stages of the pandemic and laboratory confirmation was dependent on local availability of PCR testing.

### 2.3 Variables

Patient questionnaires for adults were developed by a multidisciplinary team of researchers, clinicians and psychologists through a series of meetings and e-mail iterations [21]. These were piloted in three countries before being finalised. The UK version was piloted with patients at sites in Liverpool and Glasgow. Patient questionnaires were designed to allow self-assessment via post, or clinician-led follow up via telephone, or in outpatient clinic, to support wide dissemination. All surviving patients who consented to be contacted following discharge, and for whom a valid address or phone number were provided, were contacted. Questionnaires were posted from the Outbreak Laboratory coordinating centre at the University of Liverpool, UK, with a prepaid, self-addressed envelope for returning the questionnaire. A combination of postal and telephone follow-up was used to improve response rates. Those who did not respond by post and who had a valid phone number were followed up by telephone or in outpatient clinic by local study investigators. Participants completed one questionnaire as part of this study, so there were no repeat measures. Data from responses were entered onto a Research Electronic Capture (REDCap) Database system hosted at the University of Oxford and linked with data documented during the admission with acute Covid-19 for the analysis.

Explanatory variables at the time of hospital admission, including age, sex, pre-existing comorbidities, and treatment received during the hospital admission were recorded. Maximum severity of Covid-19 during the acute hospital admission with Covid-19 was classified using the WHO COVID-19 ordinal severity scale [23]. This scale comprised of 4 levels of severity which were relevant to our in-hospital cohort; level 3 - did not receive supplemental oxygen, level 4 - received supplemental oxygen, level 5 - received high flow oxygen or NIV non-invasive ventilation (HFNC, NIV), and levels 6 and 7 - received invasive mechanical ventilation or admission to critical care [23]. We also used the WHO severity scale to account for in-hospital severity in our modelling approach [23,24].

### 2.4 Outcomes

The primary outcome was self-reported recovery at 3 to 12 months following initial Covid-19 symptoms. Secondary outcomes included persistent or new symptoms, new or worsened disability assessed using the Washington Disability Group (WG) Short Form [25], breathlessness measured using the Medical Research Council (MRC) dyspnoea scale [26], fatigue measured on a 1 to 10 visual analogue scale (VAS) where zero is no fatigue and ten is worst possible fatigue, and quality of life using the EuroQol® EQ5D-5L instrument (Supplementary Appendix 2) [27]. The MRC dyspnoea scale was developed to grade the effect of breathlessness on daily activities [26]. This 5-point scale measures perceived respiratory disability, with 1 being no breathlessness and 5 being unable to undertake activities of daily living due to breathlessness [26]. The WG Short Set tool includes six questions on functioning (vision, hearing, mobility, cognition, self-care, communication) [25]. These questions reflect a bio-psychosocial model of disability by describing level of disability and probe aspects of disability which may limit an individual's participation in society. This tool has been shown to detect the majority of disabilities and is standardised for use globally [25]. The EuroQol®EQ5D-5L tool was used to measure psychosocial health and quality of life [27]. The tool covers five dimensions: mobility, self-care, usual activities, pain/discomfort and anxiety/depression. The person indicates his/her health state for each of the five dimensions. To compare the change in EQ5D-5L at the time of post-hospital follow-up to before Covid-19 onset, we asked patients the same questions contained in the EQ5D-5L with the tense altered to ask specifically about pre-Covid-19 state.

### 2.5 Statistical methods

Categorical data were summarised as frequencies and percentages, and continuous data as median, alongside the corresponding interquartile range (IQR) presented as the 25^th^ and 75^th^ centile values. To test for differences across comparison groups in categorical data, we used Fisher's exact test and for continuous data, used the Wilcoxon rank-sum test for two-sample testing and Kruskal-Wallis where there were more than 2 groups. Analysis of symptom co-occurrence was done using the Jaccard similarity index and represented visually as heatmaps with dendrograms constructed from complete hierarchical clustering results (where 0 is no co-occurrence and 1 is perfect co-occurrence). We then identified clusters of symptoms based upon the hierarchical dendrograms and clusters that were seen on the heatmap.

For disability, breathlessness, and EQ5D-5L index (health state), we calculated the change in value reported by participants before onset of their Covid-19 illness compared to the follow up assessment. For health state at the follow up assessment, we used the EQ5D-5L with the English standardised valuation study protocol (EQ-VT) value set, developed by the EuroQol group on the composite time trade-off (cTTO) valuation [27]. Overall changes in summary health index, before and after Covid-19 onset, were summarised for the cohort using the Paretian Classification of Health Change (PCHC) method [28,29]. Summary EQ5D-5L indices and change in summary EQ5D-5L index were measured on a scale of 0 to 1, with 1 being perfect health and 0 being worst health imaginable. We calculated both the overall estimates and estimates for individual EQ5D-5L dimensions. As a sensitivity analysis, to identify the impact of the presence of a positive SARS-CoV-2 PCR test, we excluded those participants who did not have a positive PCR test reported. We then looked at the outcomes and ran the same models for the whole cohort in this subgroup to ensure there was no change in the direction or magnitude of the effect.

We created models to adjust for age, sex, presence of comorbidities and in-hospital severity of Covid-19, according to the maximum level of respiratory support that was required. Multilevel logistic regression was used for binary outcomes, and linear regression models were used for continuous outcomes. In both model types, we adjusted for the effects of explanatory variables using fixed-effects and centre by including a random-effects term. For all models, variable selection was performed based on clinical plausibility, and final models were selected based on clinical relevance guided by minimisation of the Akaike information criterion (AIC). Variables were only included in the model if they were present during the first hospital admission for Covid-19. All models were checked for first order interactions and any meaningful interactions were retained and incorporated as dummy variables. Effect estimates are presented as odds ratios for binary outcomes or mean differences for continuous outcomes, alongside the corresponding 95% confidence interval (95% CI). Statistical analyses were performed using R version 3.6.3 (R Foundation for Statistical Computing, Vienna, AUT) with the tidyverse, finalfit, eq5d and Hmisc packages. Statistical significance was taken at the level of P ≤ 0.05.

### 2.6 Public and patient involvement

This was an urgent public health research study in response to a public health emergency of international concern. Patients and the public were therefore not involved in the design, of the acute phase rapid response research. However, patients and people living with long Covid were involved in the design, conduct and interpretation of the follow up study. The follow up data collection survey and associated patient information was informed by the founding members of the Long Covid support group, who themselves are living with long Covid. The survey was also piloted in several settings in the UK with patients affected by Covid-19 from different demographics, and feedback incorporated into the final version. This included suggestions on the data on symptoms collected and the way questions were asked as well as on the patient information. The results and interpretation of the findings and final manuscript were informed by members of the Long Covid support group.

### 2.7 Role of the funding source

The study sponsors and funders had no role in the study design, collection, analysis, interpretation of data, writing of the report, or the decision to submit the article for publication. Investigators were independent from funders and the authors have full access to all of the data, including any statistical analysis and tables.

## 3 Results

**Fig. 1:**
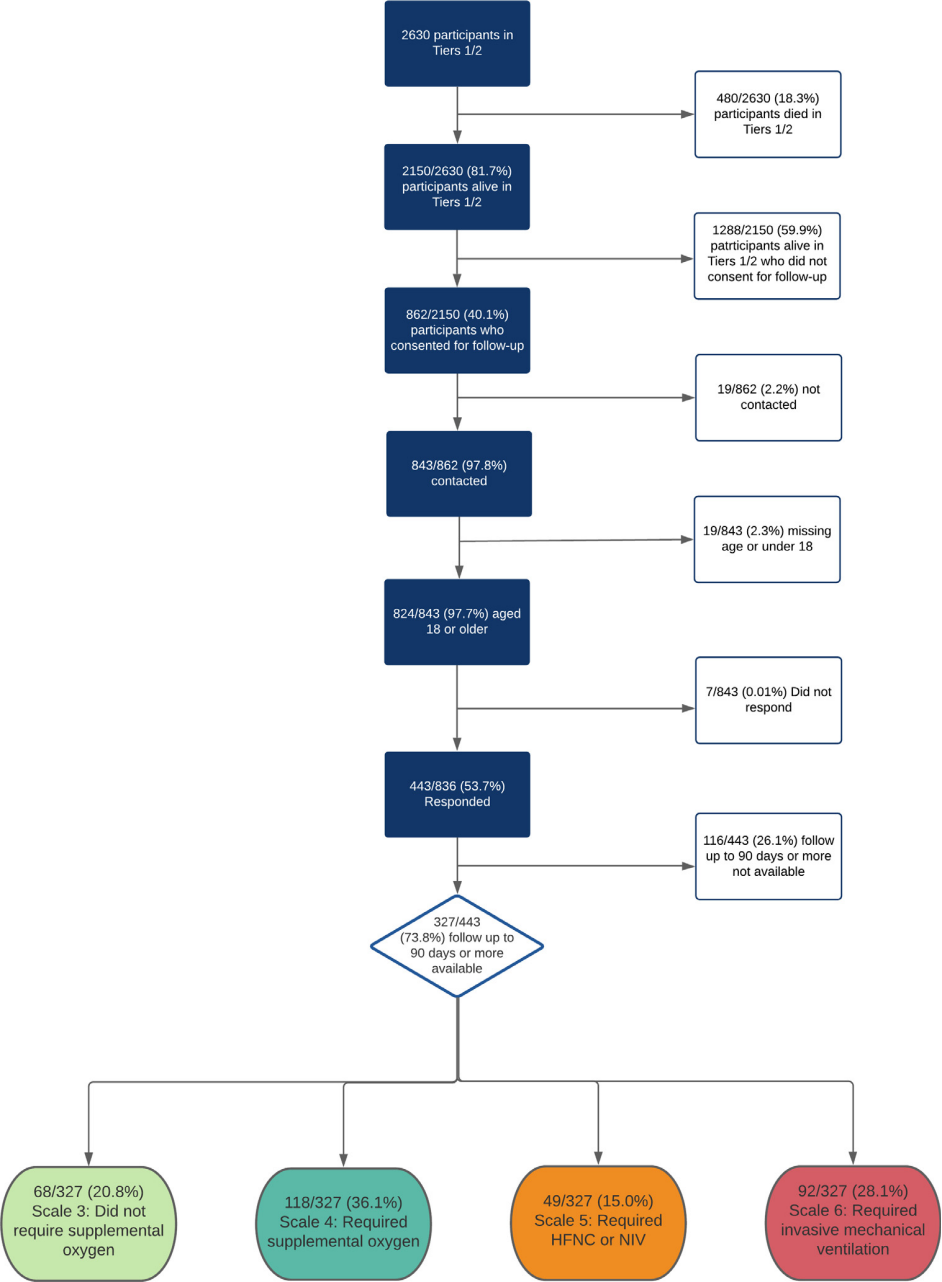
Patient inclusion flowchart

Of the 2150 eligible people in the CCP-UK study who were discharged from their acute admission alive, 40•1% (862/2150) provided consent to be contacted for follow-up. Of these, 97•8% (843/862) were contacted. From these 843 people, 97•7% (824/843) were 18 or over and 53•7% (443/824) completed the follow-up questionnaire. Finally, of respondents 73•8% (327/443) responded 90 days or more after symptom onset. Included participants completed the follow-up questionnaire through self-assessment (71·6% 234/327), telephone (24·5% 80/327) or in outpatient clinic (4·0% 13/327, Fig. 1). The median follow-up time from symptom onset was 222 days (IQR: 189 to 269 days, range: 112 to 343 days, Table 1).

**Table 1:** Characteristics of participants who responded

		Recovered or unsure	Does not feel fully recovered	(Missing)	p-value
Total N (%)		144 (44• 0)	179 (54·7)	4 (1·2)	
Age	Median (IQR)	60·5 (53·2 to 69.8)	59·4 (50·3 to 66·7)	54·2 (48·2 to 58·5)	0·089
	Under 50	26 (37·1)	43 (61·4)	1 (1·4)	
	50 to 69	83 (42·6)	109 (55·9)	3 (1·5)	
	Over 70	35 (56·5)	27 (43·5)	0 (0·0)	
Sex at Birth	Male	87 (45·3)	103 (53·6)	2 (1·0)	0·683
	Female	57 (42·2)	76 (56·3)	2 (1·5)	
Ethnicity	White	115 (43·4)	147 (55·5)	3 (1·1)	0·206
	South Asian	2 (25·0)	6 (75·0)	0 (0·0)	
	East Asian	3 (75·0)	1 (25·0)	0 (0·0)	
	Black	10 (66·7)	5 (33·3)	0 (0·0)	
	Other Ethnic Minority	10 (47·6)	10 (47·6)	1 (4·8)	
	(Missing)	4 (28·6)	10 (71·4)	0 (0·0)	
Smoking	Never Smoked	84 (47·7)	90 (51·1)	2 (1·1)	0·892
	Current Smoker	4 (57·1)	3 (42·9)	0 (0·0)	
	Former Smoker	43 (46·7)	47 (51·1)	2 (2·2)	
	(Missing)	13 (25·0)	39 (75·0)	0 (0·0)	
Diabetes	No	112 (44·1)	138 (54·3)	4 (1·6)	0·972
	Yes	27 (43·5)	35 (56·5)		
Obesity (as defined by clinical staff)	No	116 (45·7)	136 (53·5)	2 (0·8)	0·291
	Yes	20 (35·7)	34 (60·7)	2 (3·6)	
	(Missing)	8 (47·1)	9 (52·9)	0 (0·0)	
Chronic cardiac disease	No	119 (43·8)	149 (54·8)	4 (1·5)	1·000
	Yes	20 (45·5)	24 (54·5)	0 (0·0)	
	(Missing)	5 (45·5)	6 (54·5)	0 (0·0)	
Chronic pulmonary disease (not asthma)	No	126 (43·4)	160 (55·2)	4 (1·4)	0·592
	Yes	12 (52·2)	11 (47·8)	0 (0·0)	
	(Missing)	6 (42·9)	8 (57·1)	0 (0·0)	
Asthma (physician diagnosed)	No	114 (45·2)	135 (53·6)	3 (1·2)	0·410
	Yes	25 (38·5)	39 (60·0)	1 (1·5)	
	(Missing)	5 (50·0)	5 (50·0)	0 (0·0)	
Chronic kidney disease	No	131 (44·0)	163 (54·7)	4 (1·3)	1·000
	Yes	8 (44·4)	10 (55·6)	0 (0.0)	
	(Missing)	5 (45·5)	6 (54·5)	0 (0·0)	
Malignant neoplasm	No	136 (44·4)	166 (54·2)	4 (1·3)	1·000
	Yes	4 (40·0)	6 (60·0)	0 (0·0)	
	(Missing)	4 (36·4)	7 (63·6)	0 (0·0)	
Rheumatologic disorder	No	131 (45·2)	155 (53·4)	4 (1·4)	0·334
	Yes	8 (33·3)	16 (66·7)	0 (0·0)	
	(Missing)	5 (38·5)	8 (61·5)	0 (0•0)	
ISARIC4C Mortality Score (predicted in hospital mortallity)	Median (IQR)	7·0 (5·0 to 9·0)	6·0 (4·0 to 9·0)	5·5 (5·0 to 6·0)	0·648
Severity	Scale 3 (did not receive supplemental oxygen)	34 (50·0)	33 (48·5)	1 (1·5)	0·001
	Scale 4 (received supplemental oxygen)	63 (53·4)	53 (44·9)	2 (1·7)	
	Scale 5 (received HFNC or NIV)	22 (44·9)	27 (55·1)	0 (0·0)	
	Scale 6 or 7 (received invasive mechanical ventilation or critical care)	25 (27·2)	66 (71·7)	1 (1·1)	
Critical care admission	Ward level care only	99 (50·3)	96 (48·7)	2 (1·0)	0·008
	Admitted to Critical Care	45 (34·6)	83 (63·8)	2 (1·5)	
Length of stay (days)	Median (IQR)	8·0 (5·0 to 13·0)	11·0 (6·2 to 25·0)	4·5 (3·0 to 7·8)	<0·001
Time from symptoms to completing survey (days)	Median (IQR)	221·0 (190·0 to 245·0)	224·0 (188·0 to 292·0)	210·0 (194·2 to 218.·5)	0·419
Time from discharge to completing survey (days)	Median (IQR)	200·0 (177·0 to 230·0)	199·0 (161·0 to 268·0)	195·0 (178·0 to 208·2)	0·846

HFNC – High flow nasal cannulae, NIV – Noninvasive ventilation, IQR – Interquartile range, presented as 25^th^ to 75^th^ centiles. Numbers are presented as N (%), unless otherwise denoted as a continuous variable.

### 3.1 Participant characteristics

Table 1 shows the characteristics of participants who responded. The majority of participants were male (58·7%, 192/327), with a median age of 59·7 (25^th^ centile 51.7 to 75^th^ centile 67·7) years and of white ethnicity (81·0%, 265/327, Table 1). Asthma (19·9%, 65/327) and diabetes (19·0%, 62/327) were the most common comorbidities (Table 1). 28•1% (92/327) received invasive mechanical ventilation. Compared with the study population who were contacted and did not respond, respondents were significantly more likely to be of white ethnicity (81·0%, 265/327 participants versus 66·6%, 331/544 of non-respondents), were more likely to be ex-smokers (28·1%, 92/327 participants versus 24·7% 123/497 of non-respondents) and were more likely to have been admitted to critical care (39·8% 130/327 in participants versus 26·8% 133/497 in non-respondents, Supplementary Table 1).

### 3.2 Outcomes and symptoms

**Fig. 2:**
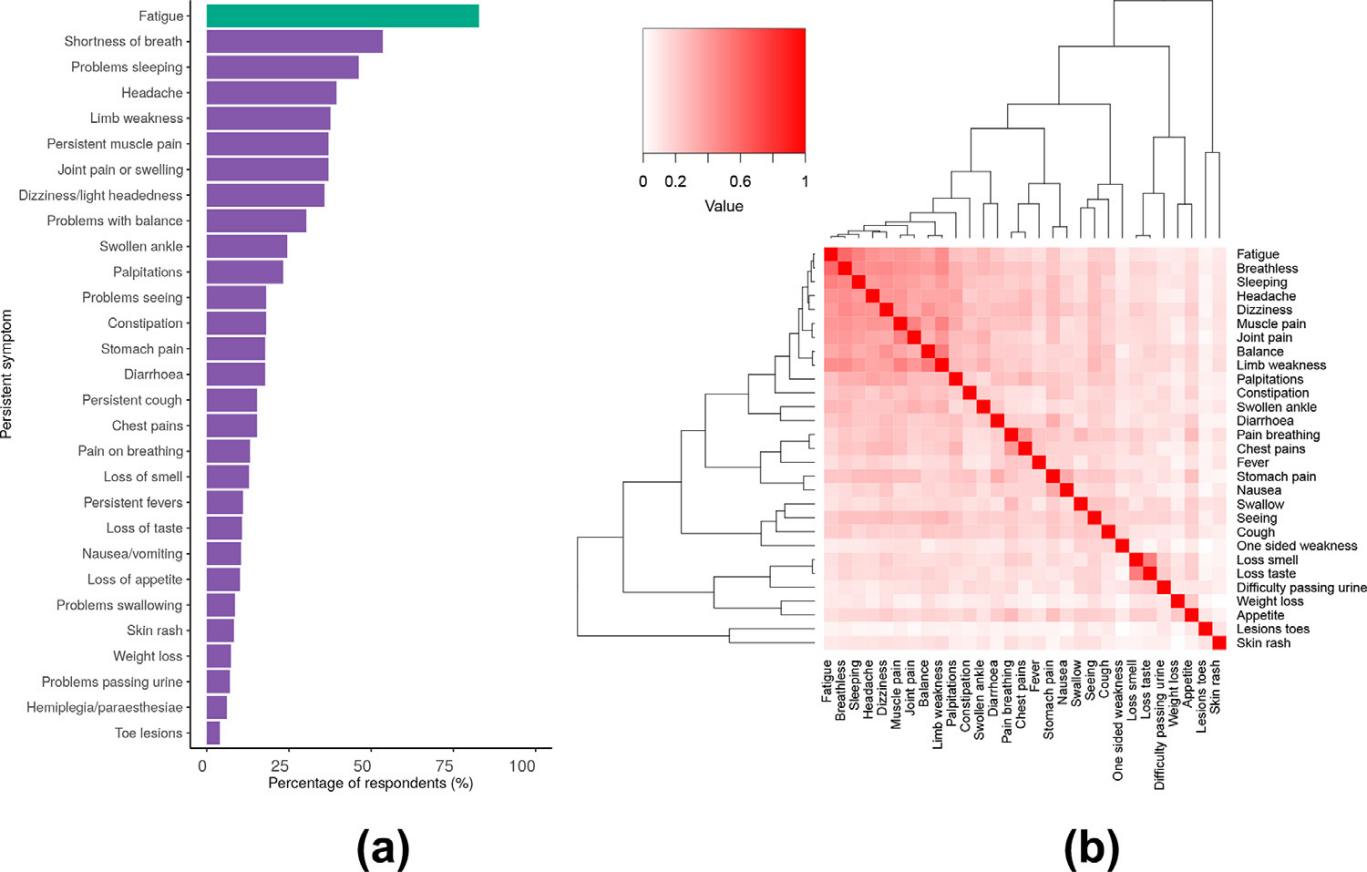
Proportion of new or persistent symptoms occurring (Fig. 2A) and their co-occurrence with each other (Fig. 2B). For Figure 2A, fatigue is coloured in green as this outcome was derived from the fatigue visual analogue outcome, where a fatigue rating of 2 or greater was considered as the presence of the fatigue symptom (see Supplementary Table 7 for raw values). Erectile dysfunction affected 23·4% (45/192) of males included, not shown as Figure 2A presents data for any sex. For Figure 2B, the Jaccard similarity index was calculated and presented as intensity of red colour, with 0 (white) being no co-occurrence and 1 (bright red) being always co-occurring.

Of 327 participants, 54·7% (179/327) did not feel they had fully recovered at the time of follow-up. At the univariable level, there were no associations between not feeling recovered and the risk factors of age, sex, ethnicity, and comorbidities (Table 1) but we found patients with a higher severity of acute disease were significantly more likely not to feel recovered. Persistent or new symptoms were reported by 93·3% (305/327) participants (Table 2). The most frequently reported symptoms were fatigue 82·8% (255/308), shortness of breath 53·5% (175/327), and problems sleeping 46·2% 151/327, Fig. 2A).

**Table 2:** Long-term outcomes by severity of acute Covid-19

		Scale 3 (did not receive supplemental oxygen)	Scale 4 (received supplemental oxygen)	Scale 5 (received HFNC or NIV)	Scale 6 or 7 (received invasive mechanical ventilation or critical care)	p-value
Total N (%)		68 (20·8)	118 (36·1)	49 (15·0)	92 (28·1)	
Self-reported overall recovery	Feels fully recovered	21 (30·9)	34 (28·8)	11 (22·4)	17 (18·5)	0·006
	Does not feel fully recovered	33 (48·5)	53 (44·9)	27 (55·1)	66 (71·7)	
	Not sure	13 (19·1)	29 (24·6)	11 (22·4)	8 (8·7)	
	(Missing)	1 (1·5)	2 (1·7)	0 (0·0)	1 (1·1)	
New or persistent symptoms	No new or persistent symptoms	3 (4·4)	9 (7·6)	6 (12·2)	4 (4·3)	0·268
	New or persistent symptoms	65 (95·6)	109 (92·4)	43 (87·8)	88 (95·7)	
Change in breathlessness after Covid-19 (MRC Dyspnoea)	No change	25 (36·8)	55 (46·6)	20 (40·8)	27 (29·3)	0·062
	Less breathless	2 (2·9)	5 (4·2)	2 (4·1)	2 (2·2)	
	More breathless	32 (47·1)	41 (34·7)	24 (49·0)	56 (60·9)	
	(Missing)	9 (13·2)	17 (14·4)	3 (6·1)	7 (7·6)	
Fatigue level (0 to 10 VAS)	Median (IQR)	5·5 (2·0 to 7·0)	4·0 (2·0 to 7·0)	5·0 (2·0 to 7·0)	5·0 (2·0 to 7·0)	0·469
EQ5D-5L change in overall summary index	Median (IQR)	-0·1 (-0·2 to 0·0)	-0·0 (-0·1 to 0·0)	-0.1 (-0·2 to 0·0)	-0·1 (-0·3 to -0·0)	0·004
Washington Group Short Set	No change in disability	52 (76·5)	88 (74·6)	35 (71·4)	66 (71·7)	0·892
	New or worse	15 (22·1)	27 (22·9)	13 (26·5)	24 (26·1)	
	(Missing)	1 (1·5)	3 (2·5)	1 (2·0)	2 (2·2)	

HFNC – High flow nasal cannulae, NIV – Noninvasive ventilation, MRC – Medical Research Council, IQR – Interquartile range, presented as 25^th^ to 75^th^ centiles. Numbers are presented as N (%), unless otherwise denoted as a continuous variable.

A heatmap and dendrogram of symptom co-occurrence identified two major clusters of symptoms (Fig. 2B); a fatigue, myalgia and sensorineural deficits cluster and an olfactory, appetite and urinary cluster (loss of smell, loss of taste, difficulty passing urine, weight loss and loss of appetite). Within the fatigue, myalgia and sensorineural deficits cluster, there was a distinct minor cluster affecting movement (muscle pain, joint pain, balance and limb weakness).

**Fig. 3:**
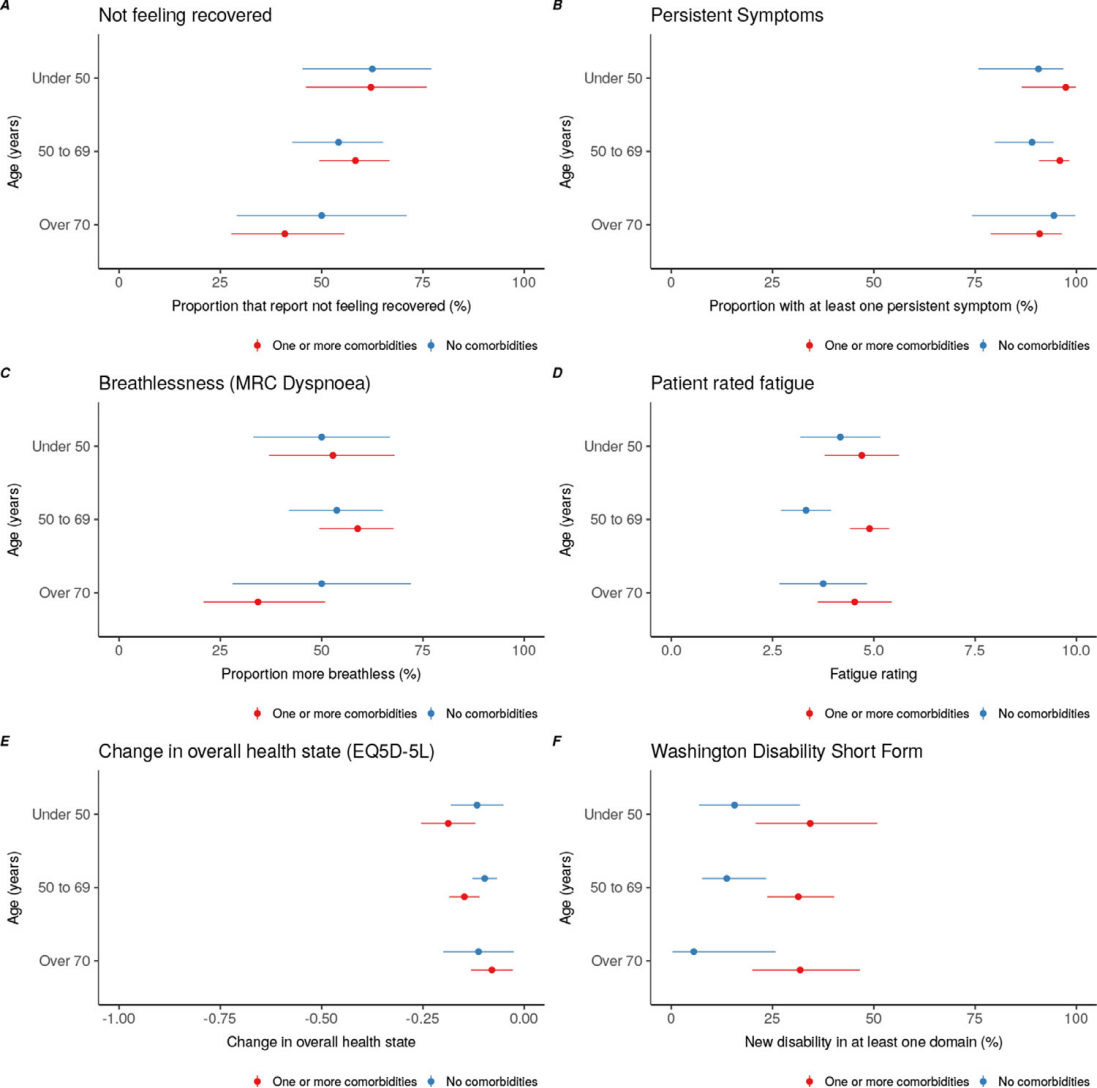
Outcomes stratified by age and presence of one or more comorbidities. Figure 3A – Proportion of participants not feeling fully recovered; Figure 3B – Proportion of participants with new or persistent symptoms; Figure 3C - Proportion of participants with increased breathlessness as measured by MRC dyspnoea scale; Figure 3D – Participant rated fatigue on 0 to 10 VAS; Figure 3E – Change in overall EQ5D-5L summary health index; Figure 3F – presence of new or worse disability in at least one Washington Group disability domain. Point estimates presented alongside 95% confidence intervals. MRC – Medical Research Council, VAS – Visual Analogue Scale.

**Fig. 4:**
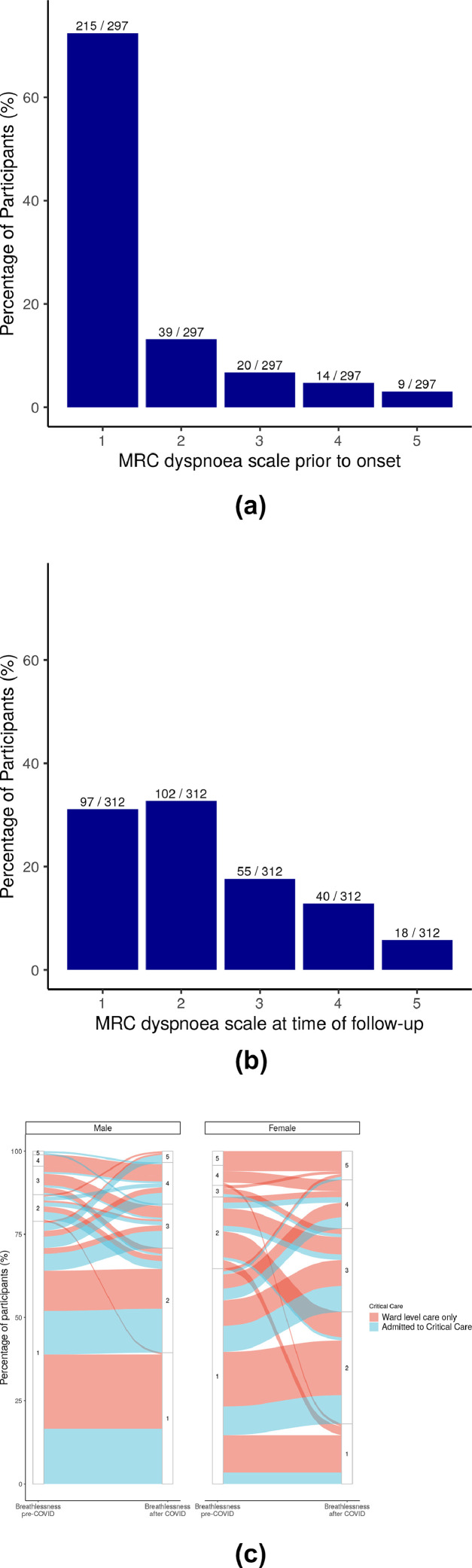
MRC Dyspnoea scale prior to Covid-19 onset and at the time of follow-up. Figure 4A – MRC dyspnoea scale reported prior to onset of Covid-19 symptoms; Figure 4B – MRC dyspnoea scale at the time of follow-up; Figure 4C – Alluvial plot of proportion of the changes in proportion of males and females in each MRC scale grade before symptom onset and at time of follow-up, stratified in each sex group by admission to critical care. In Figure 4C, for females, there are greater numbers of participants who begin at MRC 1 and transition to higher levels on the scale compared with males. MRC – Medical Research Council

In addition to symptomatic breathlessness, 46·8% (153/327) of participants reported increased breathlessness compared to their pre-Covid-19 baseline. Overall, change in breathlessness was not affected by age or number of comorbidities (Fig. 3), but was significantly higher in females compared to males (41·7%, 80/192 in males versus 54·1% 73/135 in females). Of participants with a pre-Covid-19 MRC grade 1, 34·0% (73/215) reported an increase to grade 2, and 25·6% (55/213) reported an increase to grades 3-5 at time of post hospital follow-up (Fig. 4A to 4C). Proportionally, those who were admitted to critical care were more likely to have a higher MRC dyspnoea grade at the time of post hospital follow-up.

**Fig. 5:**
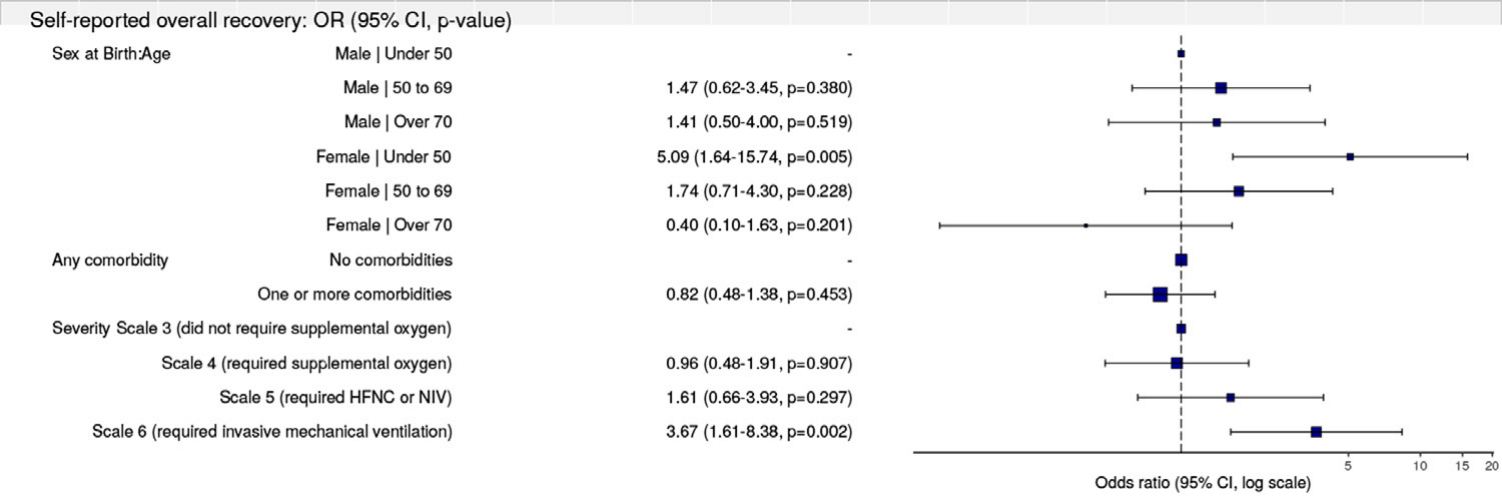
Multilevel model for primary outcome of self-reported recovery (reference level is feeling fully recovered)

Overall, intensity of fatigue was unrelated to age or disease severity in hospital (Fig. 3, Table 2), but females were found to have significantly increased levels of fatigue compared with males (median fatigue 0-10 VAS score, males 4·0, IQR 2.0 to 6; versus females 6·0, IQR 2·0 to 7·0, Supplementary Table 2, Supplementary Figure 1).

New or worsened disability in at least one Washington Group domain was experienced by 24·2% (79/327). This did not change by in-hospital Covid-19 severity (Table 2) or comorbidities (Fig. 3). Females reported a greater number of new or worsened disabilities compared to males (20·3%, 39/192 in males compared with 29·6%, 40/135 in females, Supplementary Table 2). The most affected domain was walking and mobility (33·3% 109/327 new mild or worsened disability, 6·4% 21/327 new moderate or worsened disability and 0·3% 1/327 new severe or worsened disability, Supplementary Table 3), followed by memory and concentration (30·0% 90/327 new mild or worsened disability, 9.8% 32/327 new moderate or worsened disability). There were significant differences in domains affected by sex, with females reporting significantly higher levels of visual disabilities (12·0% 23/192 new mild or worsened disability for males versus 25·2% 34/135 new mild or worsened disability for females, Supplementary Table 3), higher levels of walking disability (28.6% 55/192 new mild or worsened disability for males versus 40·0% 54/135 new or worsened mild disability for females; 5·6% 11/192 new moderate or worsened disability for males versus 7·4% 10/327 new moderate or worsened disability for females) and memory disability (27·1% 52/192 new mild or worsened disability for males versus 34·1% 46/135 new mild or worsened disability for females; 7·3% 14/192 new moderate or worsened disability for males versus 13·3% 18/135 new moderate or worsened disability for females, Supplementary Table 4).

Overall summary EQ5D-5L index was 10% lower overall following Covid-19 (median difference -0·1 points, -0·2 25^th^ centile to 0·0 75^th^ centile, Table 2). This change was independent of age or comorbidities (Fig. 3). The EQ5D-5L dimensions for which most participants reported worsening were usual activities (38·9%, 121/311), anxiety/depression (37·6%, 117/311), and pain/discomfort (37·6%, 117/311) (Supplementary Table 5). Female sex was significantly associated with increased problems in the usual activity, pain or discomfort and anxiety and depression domains (Supplementary Table 6).

### 3.3 Predictors of long-term Covid-19 outcomes

Using multilevel regression models, we adjusted for the effects of age by sex (as this was identified as a significant interaction and retained in our models), the presence of comorbidity and initial in-hospital severity of Covid-19. This generated 6 groups; Males under 50 (34/327), males between 50 and 69 (114/327), males 70 and over (44/327), females under 50 (36/327), females between 50 and 69 (81/327), and females 70 and over (18/327). For the primary outcome of self-reported overall recovery, females under 50 were 5 times less likely to feel fully recovered (Fig. 4). Similarly, those who received invasive mechanical ventilation were 3·6 times less likely to feel fully recovered (Fig. 4). For the secondary outcomes, age did not appear to be associated with better or worse long-term outcomes (Table 3). Females under 50 were more likely than men to experience persistent fatigue and seven times more likely to experience greater breathlessness, twice as likely to develop new disability and had a significantly poorer health state (EQ5D-5L), all of which persisted in adjusted analyses (Table 3). Participants with one or more comorbidities were more likely to experience greater fatigue, disability, and a poorer health state (EQ5D-5L, Table 3).

**Table 3:** Multilevel regression models for secondary outcomes of new or persistent symptoms, change in MRC dyspnoea scale, fatigue, EQ5D-5L summary index change and Washington Short Set new or worse disability.

Explanatory variable		New or persistent symptoms: OR (95% Confidence Interval)	Change in MRC Dyspnoea: OR (95% Confidence Interval)	Fatigue level: Coefficient (95% Confidence Interval)	EQ5D-5L summary index change: Coefficient (95% Confidence Interval)	Washington Short Set new or worse disability: OR (95% Confidence Interval)
Sex at Birth by Age	Male | Under 50	-	-	-	-	-
	Male | 50 to 69	0·82 (0·21-3·30, p=0·783)	2·20 (0·89-5·45, p=0·088)	0·44 (-0·56 to 1·44, p=0·194)	-0·05 (-0·11 to 0·02, p=0·093)	1·66 (0·51-5·42, p=0·401)
	Male | Over 70	0·74 (0·14-3·83, p=0·720)	2·59 (0·84-7·95, p=0·096)	0·38 (-0·84 to 1·60, p=0·272)	-0·04 (-0·12 to 0·04, p=0·184)	2·08 (0·55-7·96, p=0.283)
	Female | Under 50	2·75 (0·26-28·92, p=0·400)	7·15 (2·24-22·83, p=0·001)	2·06 (0·81 to 3·31, p=0·001)	-0·19 (-0·27 to -0·11, p<0·001)	4·22 (1·12-15·94, p=0·034)
	Female | 50 to 69	2·10 (0·39-11·37, p=0·389)	6·18 (2·28-16·78, p<0·001)	1·20 (0·15 to 2·24, p=0·012)	-0·10 (-0·17 to -0·03, p=0·003)	2·70 (0·81-9·03, p=0·107)
	Female | Over 70	1·21 (0·11-13·89, p=0·876)	0·62 (0·12-3·11, p=0·562)	0·29 (-1·33 to 1·92, p=0·362)	-0·06 (-0·17 to 0·04, p=0·109)	1·88 (0·36-9·82, p=0·452)
Any comorbidity	No comorbidities	-	-	-	-	-
	One or more comorbidities	2·28 (0·92-5·65, p=0·076)	0.74 (0·42-1·31, p=0·304)	0·95 (0·35 to 1·55, p=0·001)	-0·02 (-0·06 to 0·02, p=0·139)	2·96 (1·57-5·57, p=0·001)
Severity	Scale 3 (did not receive supplemental oxygen)	-	-	-	-	-
	Scale 4 (received supplemental oxygen)	0·61 (0·15-2·43, p=0·483)	0·51 (0·24-1·07, p=0·076)	-0·26 (-1·06 to 0·55, p=0·266)	0·04 (-0·01 to 0·09, p=0·077)	1·11 (0·51-2·40, p=0·798)
	Scale 5 (received HFNC or NIV)	0·32 (0·07-1·46, p=0·142)	0·89 (0·36-2·21, p=0·794)	-0·20 (-1·22 to 0·83, p=0·354)	0·01 (-0·06 to 0·08, p=0·371)	1·32 (0·49-3·51, p=0·583)
	Scale 6 or 7 (received invasive mechanical ventilation or critical care)	1·18 (0·24-5·95, p=0·838)	1·82 (0·79-4·22, p=0·162)	-0·18 (-1·09 to 0·74, p=0·354)	-0·05 (-0·11 to 0·02, p=0·073)	1·48 (0·63-3·52, p=0·370)

HFNC – High flow nasal cannulae, NIV – Noninvasive ventilation, MRC – Medical Research Council. Model metrics: For persistent symptoms - Number in model = 327, Number of groups = 32, AIC = 172·4, C-statistic = 0·683; For change in MRC dyspnoea level - Number in model = 291, Number of groups = 32, AIC = 383.1, C-statistic = 0.767; For change in fatigue - Number in model = 308, Number of groups = 32, Log likelihood = -724·13, REML criterion = 1448·3; For change in health state (EQ5D-5L) - Number in model = 311, Number of groups = 32, Log likelihood = 80·55, REML criterion = -161⋅1; For change in Washington short set disability - Number in model = 320, Number of groups = 31, AIC = 355·1, C-statistic = 0·74.

To explore these findings further, we then looked to see if there were any differences in comorbidity or in-hospital disease severity by sex. We found males were significantly more likely to have greater comorbidity (Supplementary Table 8) and more severe in hospital disease (Supplementary Table 9).When we performed a sensitivity analysis for our overall findings, this time excluding patients who did not have a positive SARS-CoV-2 PCR test. The results of these sensitivity analyses (Supplementary Tables 10 and 11), show no effect of SARS-CoV-2 positivity on our estimates for long-term outcomes.

## 4 Discussion

We found high rates of long-term symptoms and poor long-term outcomes, which were present several months after hospitalisation for Covid-19. This has implications for planning of care and rehabilitation pathways. These patients may present to multiple specialities within the health care system unless coordinated by a dedicated long Covid service. The range of syndromes identified highlights a need for long Covid clinics to triage patients for further comprehensive diagnostics, based on symptom cluster, including specialist imaging, for assessing underlying aetiology to inform treatment and improve outcomes. Females under 50, and those with severe acute disease requiring critical care had the worst long-term outcomes even after adjusting for severity of the initial illness. Interestingly, our findings were largely unaffected by existing patient comorbidities or disability.

Our findings add considerably to the current literature, as we identify the main risk factor for worse long-term outcomes are being female and under the age of 50. We also have been able to quantify the significant deterioration in disability and breathlessness-related disability in detail. The range of symptoms reported include those which may be related to direct lung damage, such as breathlessness, and also those for which an underlying pathophysiological mechanism may be less clear such as fatigue, muscle pain and cognitive complaints. The latter group are also features of other post infectious syndromes and post intensive care syndrome, and may have a similar aetiology, such as infection triggered autoimmunity, dysautonomia or other mechanism [6], [7], [8], [9], [10]. Our study did not make laboratory measurements or collect biological samples as part of the follow-up and hence is not designed to elucidate mechanisms. Future studies which do this will be key to identifying relevant therapeutic targets in long-Covid.

Many of our findings are largely in agreement with other recent studies in other populations globally, which also found high rates of breathlessness and fatigue [4,5]. In the community setting, a recent mobile application-based study, described very high rates of breathlessness (71%) and fatigue (98%) in those reporting symptoms persisting over 28 days [2]. Interestingly, in our population, the presence of symptoms many months after initial infection are higher than the 76% reported by Huang et al. and three times higher than that reported by Munblit et al. There are several reasons why we have found higher rates, which could be related to those responding to each study, or the severity of disease across the different study populations. The Huang et al. and Munblit et al. studies included very small numbers of patients requiring critical care or mechanical ventilation (1% in Huang et al. and under 2·6% in Munblit et al, in contrast to 28·1% 92/327 in our study), suggesting there are significant differences between these populations and our study population; Survivors of general critical illness, independent of baseline disease, may experience persistent breathlessness, fatigue, muscle weakness, and other symptoms of post-intenisve care syndrome which cause substantial decifits in quality of life that may persist for many years [10]. There may several reasons for this difference in study population, such as challenges to recruitment of critically unwell patients, differing in-hospital mortality rates, preexisting population comorbidities or pressure on the healthcare systems during the pandemic. Based on data from several countries, the higher rates of participants requiring critical care in our study suggests our data is likely to be more generalisable [30], [31], [32], [33].

In our study, being young, female and having a high severity of acute disease were the strongest independent predictors of poor long-term outcomes. It is unclear why females had the worst outcomes. This could be to do with the effects of initial exposure, where females are more likely to be in industries where exposure to SARS-CoV-2 may be higher [34], however recent data suggests teachers do not have greater exposure than other working-age populations and there is emerging evidence of divergent host responses to SARS-CoV-2 infection [35,36]. Another explanation is that females are more likely to survive severe acute disease than men, so could have worse long-term outcomes as a result. However, in our data, we could not find any differences by sex across several measures of disease severity. A further possibility is that men felt less able or inclined to disclose symptoms. There is some discussion in the literature that recall bias, and also reporting of symtoms may differ between males and females, which may account for some of the gender difference seen – however this would not account for these differences being also related to age as well as gender [37]. From our findings it is clear more research is required into why females have worse long-term outcomes, particularly as sectors where females are likely to have greater exposure to SARS-CoV-2 are beginning to reopen (e.g. education, hospitality and healthcare).

There are several limitations to our study. First, we were not able to follow all the cases that were discharged from hospital, either because they did not give permission or because they did not respond to repeated requests for information. We attempted to reach non-responders to the survey via telephone follow-up to limit potential for selection bias, but not all could be reached. However the 73% response rate is substantially above the expected response rate for multi-modal questionaires (60%) [38]. It is possible that those who did not respond might have been well and therefore uninterested in responding, but it could also be that some were too unwell to respond, had died or moved away. Our results may therefore not be fully representative of the entire population of those hospitalised with Covid-19. The potential completion bias may have led to over representation of the prevalence of people affected by long Covid, but may also be underrepresenting people from different demographics. Nevertheless, the data shows that amongst the cohort of people that consented to be contacted post Covid-19 hospital discharge, a large proportion were not fully recovered from Covid more than three months post discharge. This is similar to what has been reported in other countries [4,5].

Secondly, we did not include patients hospitalised with other non-Covid-19 illness or a contemporaneous control group, therefore it is unknown if the changes in our outcomes e.g. quality of life, are specific to recovery from Covid-19 or may be linked to other aspects of life during the pandemic. The study used to generate this data (ISARIC WHO Clinical Characterisation Protocol/CCP-UK) is a prospective pandemic preparedness protocol which is agnostic to disease and has a pragmatic design to allow recruitment during pandemic conditions. Thirdly, patients only completed the survey at one timepoint, limiting comparison across repeat measures. This also meant retrospective measures asking patients to rate outcomes before their Covid-19 illness were included, which are open to recall bias. Fourth, the differences in collecting data (in clinic, by post, by telephone) may add to heterogeneity in the data. Finally, as our study focussed on hospitalised patients primarily from the first wave of infection in the UK, our data cannot be generalised to those with disease managed in the community who comprise the majority of individuals affected by Covid-19.

Future research should focus on establishing the optimal care of this cohort, identifying interventions to test in randomised trials and to identify the mechanisms underlying adverse long-term outcomes. The PHOSP-Covid (Post-HOSPitalisation Covid-19) study is ongoing and will inform patient care by adding to our data on the long-term sequelae of Covid-19, looking at the impact on these of acute and post-discharge interventions, and exploring possible mechanisms including measurement of laboratory parameters and functional diagnostics [39].

## 5 Conclusion

In our study of 327 patients who were discharged alive from hospital, we found most participants reported symptoms months after acute Covid-19 infection. The most common symptoms were fatigue and breathlessness. Participants reported significant difficulties, including increased breathlessness, new or worsened disability and worse quality of life following Covid-19. These symptoms were largely independent of age and prior comorbidity, suggesting that the long-term effects of Covid-19 are determined by factors that differ from those that predict increased mortality. Moreover, the high frequency and severity of long-term symptoms emphasise the importance of long-Covid symptoms and the potential long-term impact on population health and wellbeing. The data highlights an urgent need for access to comprehensive assessments for people living with long Covid, including complex diagnostics to identify aetiology and inform appropriate treatment to improve long term Covid-19 outcomes.

^ISARIC Global Covid-19 follow up working group:

Adam Ali, John H Amuasi, Andrea Angheben, John Kenneth Baille, Valeria Balan, Ibrahim Richard Bangura, Anna Beltrame, Frank Bloos, Lucille Blumberg, Fernando Bozza, Danilo Buonsenso, Caterina Caminiti, Gail Carson, Daniel Cassaglia, Muge Cevik, Allegra Chatterjee, Andrew Dagens, Yash Doshi, Thomas M. Drake, Murray Dryden, Anne Margarita Dyrhol Riise, Michael Edelstein, Rob Fowler, Kyle Gomez, Katrina Hann, Ewen M Harrison, Madiha Hashmi, Lars Hegelund, Aquiles Henriquez Trujillo, Antonia Ho, Jan Cato Holter, Jane Ireson, Nina Jamieson, Waasila Jassat, Edwin Jesudason, Anders Benjamin Kildal, Sulaiman Lakoh, Nicola Latronico, James Lee, Wei Shen Lim, Sam Lissaeur, Nazir Lone, David J Lowe, Sinnadurai Manohan, Romans Matulevics, Joanne McPeake, Laura Merson, Roberta Meta, Melina Michelen, Sarah Moore, Ben Morton, Caroline Mudara, Daniel Munblit, Srinivas Murthy, Behzad Nadjm, Ebrahim Ndure, Nikita Nekliudov, Piero Olliaro, Carlo Palmieri, Prasan K Panda, Simone Piva, Daniel R Plotkin, Matteo Puntini, Jordi Rello, Liliana Resende, Luis Felipe Reyes, Ishmeala Rigby, Sergio Ruiz Saltana, Clark D Russell, Steffi Ryckaert, Janet T Scott, Malcolm G. Semple, Louise Sigfrid, Girish Sindhwani Pulm, Arne Søraas, Renaud Tamisier, Lance Turtle, Caroline Vika, Natalie Wright

^^ISARIC4C investigators Consortium lead investigator:

J Kenneth Baillie, Malcolm G Semple, Peter JM Openshaw; Gail Carson, Benjamin Bach, Wendy S Barclay, Debby Bogaert, Meera Chand, Graham S Cooke, Annemarie B Docherty, Jake Dunning, Ana da Silva Filipe, Tom Fletcher, Christoper A Green, Ewen M Harrison, Julian A Hiscox, Antonia Ying Wai Ho, Peter W Horby, Samreen Ijaz, Saye Khoo, Paul Klenerman, Andrew Law, Wei Shen Lim, Alexander J Mentzer, Laura Merson, Alison M Meynert, Mahdad Noursadeghi, Shona C Moore, Massimo Palmarini, William A Paxton, Georgios Pollakis, Nicholas Price, Andrew Rambaut, David L Robertson, Clark D Russell, Vanessa Sancho-Shimizu, Janet T Scott, Thushan de Silva, Louise Sigfrid, Tom Solomon, Shiranee Sriskandan, David Stuart, Charlotte Summers, Richard S Tedder, Emma C Thomson, AA Roger Thompson, Ryan S Thwaites, Lance CW Turtle, Rishi K Gupta, Carlo Palmieri, Maria Zambon, Chloe Donohue, Ruth Lyons, Fiona Griffiths, Wilna Oosthuyzen, Riinu Pius, Thomas M Drake, Cameron J Fairfield, Stephen R Knight, Kenneth A Mclean, Derek Murphy, Catherine A Shaw, Michelle Girvan, Egle Saviciute, Stephanie Roberts, Janet Harrison, Laura Marsh, Marie Connor, Sophie Halpin, Clare Jackson, Carrol Gamble, Andrew Law, Murray Wham, Sara Clohisey, Ross Hendry, James Scott-Brown, Victoria Shaw, Sarah E McDonald, Jane A Armstrong, Milton Ashworth, Innocent G Asiimwe, Siddharth Bakshi, Samantha L Barlow, Laura Booth, Benjamin Brennan, Katie Bullock, Benjamin WA Catterall, Jordan J Clark, Emily A Clarke, Sarah Cole, Louise Cooper, Helen Cox, Christopher Davis, Oslem Dincarslan, Chris Dunn, Philip Dyer, Angela Elliott, Anthony Evans, Lorna Finch, Lewis WS Fisher, Terry Foster, Isabel Garcia-Dorival, William Greenhalf, Philip Gunning, Catherine Hartley, Rebecca L Jensen, Christopher B Jones, Trevor R Jones, Shadia Khandaker, Katharine King, Robyn T. Kiy, Chrysa Koukorava, Annette Lake, Suzannah Lant, Diane Latawiec, Lara Lavelle-Langham, Daniella Lefteri, Lauren Lett, Lucia A Livoti, Maria Mancini, Sarah McDonald, Laurence McEvoy, John McLauchlan, Soeren Metelmann, Nahida S Miah, Joanna Middleton, Joyce Mitchell, Shona C Moore, Ellen G Murphy, Rebekah Penrice-Randal, Jack Pilgrim, Tessa Prince, Will Reynolds, P. Matthew Ridley, Debby Sales, Victoria E Shaw, Rebecca K Shears, Benjamin Small, Krishanthi S Subramaniam, Agnieska Szemiel, Aislynn Taggart, Jolanta Tanianis-Hughes, Jordan Thomas, Erwan Trochu, Libby van Tonder, Eve Wilcock, J. Eunice Zhang, Lisa Flaherty, Nicole Maziere, Emily Cass, Alejandra Doce Carracedo, Nicola Carlucci, Anthony Holmes, Hannah Massey, Nicola Wrobel, Sarah McCafferty, Kirstie Morrice, Alan MacLean, Daniel Agranoff, Ken Agwuh, Dhiraj Ail, Erin L. Aldera, Ana Alegria, Brian Angus, Abdul Ashish, Dougal Atkinson, Shahedal Bari, Gavin Barlow, Stella Barnass, Nicholas Barrett, Christopher Bassford, Sneha Basude, David Baxter, Michael Beadsworth, Jolanta Bernatoniene, John Berridge, Nicola Best, Pieter Bothma, David Chadwick, Robin Brittain-Long, Naomi Bulteel, Tom Burden, Andrew Burtenshaw, Vikki Caruth, David Chadwick, Duncan Chambler, Nigel Chee, Jenny Child, Srikanth Chukkambotla, Tom Clark, Paul Collini, Catherine Cosgrove, Jason Cupitt, Maria-Teresa Cutino-Moguel, Paul Dark, Chris Dawson, Samir Dervisevic, Phil Donnison, Sam Douthwaite, Ingrid DuRand, Ahilanadan Dushianthan, Tristan Dyer, Cariad Evans, Chi Eziefula, Chrisopher Fegan, Adam Finn, Duncan Fullerton, Sanjeev Garg, Sanjeev Garg, Atul Garg, Effrossyni Gkrania-Klotsas, Jo Godden, Arthur Goldsmith, Clive Graham, Elaine Hardy, Stuart Hartshorn, Daniel Harvey, Peter Havalda, Daniel B Hawcutt, Maria Hobrok, Luke Hodgson, Anil Hormis, Michael Jacobs, Susan Jain, Paul Jennings, Agilan Kaliappan, Vidya Kasipandian, Stephen Kegg, Michael Kelsey, Jason Kendall, Caroline Kerrison, Ian Kerslake, Oliver Koch, Gouri Koduri, George Koshy, Shondipon Laha, Steven Laird, Susan Larkin, Tamas Leiner, Patrick Lillie, James Limb, Vanessa Linnett, Jeff Little, Mark Lyttle, Michael MacMahon, Emily MacNaughton, Ravish Mankregod, Huw Masson, Elijah Matovu, Katherine McCullough, Ruth McEwen, Manjula Meda, Gary Mills, Jane Minton, Mariyam Mirfenderesky, Kavya Mohandas, Quen Mok, James Moon, Elinoor Moore, Patrick Morgan, Craig Morris, Katherine Mortimore, Samuel Moses, Mbiye Mpenge, Rohinton Mulla, Michael Murphy, Megan Nagel, Thapas Nagarajan, Mark Nelson, Matthew K. O'Shea, Igor Otahal, Marlies Ostermann, Mark Pais, Selva Panchatsharam, Danai Papakonstantinou, Hassan Paraiso, Brij Patel, Natalie Pattison, Justin Pepperell, Mark Peters, Mandeep Phull, Stefania Pintus, Jagtur Singh Pooni, Frank Post, David Price, Rachel Prout, Nikolas Rae, Henrik Reschreiter, Tim Reynolds, Neil Richardson, Mark Roberts, Devender Roberts, Alistair Rose, Guy Rousseau, Brendan Ryan, Taranprit Saluja, Aarti Shah, Prad Shanmuga, Anil Sharma, Anna Shawcross, Jeremy Sizer, Manu Shankar-Hari, Richard Smith, Catherine Snelson, Nick Spittle, Nikki Staines, Tom Stambach, Richard Stewart, Pradeep Subudhi, Tamas Szakmany, Kate Tatham, Jo Thomas, Chris Thompson, Robert Thompson, Ascanio Tridente, Darell Tupper-Carey, Mary Twagira, Andrew Ustianowski, Nick Vallotton, Lisa Vincent-Smith, Shico Visuvanathan, Alan Vuylsteke, Sam Waddy, Rachel Wake, Andrew Walden, Ingeborg Welters, Tony Whitehouse, Paul Whittaker, Ashley Whittington, Padmasayee Papineni, Meme Wijesinghe, Martin Williams, Lawrence Wilson, Sarah Cole, Stephen Winchester, Martin Wiselka, Adam Wolverson, Daniel G Wooton, Andrew Workman, Bryan Yates, Peter Young.

## Author contributors

JTS, LS, MGS developed the concept of the follow up study. JTS, LS, LWS, TMD, EJ, WLS, CB, DJL, MC, JMcP, NL, EMH, DM, CR, AH, LT, PB, and the ISARIC Global Covid-19 follow up working group developed the follow up protocol and methodology. ISARIC4C investigators identified participants during the acute admission and entered acute phase data. PB, CD, HH, RS, JH, AG coordinated follow up survey distribution, data entry, AG, AC, LG conducted telephone follow up. LS, JTS, GC, HH coordinated resources. LS, JTS, TMD, EP, AD, PO, EH, ABD were involved in data visualisation. TMD, EP, LS, JTS, MEO'H, PJMO, CH, CEH, JKB, ABD, MGS analysed and interpreted the data. LS, TMD, EP, JTS wrote the original draft of the manuscript. All authors reviewed, and revised the manuscript prior to submission. JTS is the guarantor.

## Declaration of Interests

CRD declares funding from the Medical Research Council, UK. JM reports a University of Cambridge Research Fellowship. WSL reports unrestricted investigator-initiated research funding from Pfizer for an unrelated multi-centre study in pneumonia, in which WSL is the CI and UK NIHR research funding for unrelated clinical trials in the fields of COVID-19, tuberculosis and community-acquired pneumonia. WSL's role on the Joint Committee on Vaccination and Immunisation (JCVI), UK and chair of COVID-19 Immunisation and as National Lead on British Thoracic Society community acquired pneumonia audit programme is unpaid and unrelated to this work. CB declares a British Heart Foundation Centre award, and a project grants from the Chief Scientist Office, Scottish Government CSO Long Term Effects and from Heart Research UK unrelated to this work. LG declares support from Pfizer & Gilead for attendance at an educational meeting in Nov 2018 and April 2019, for cost of conference registration fee, accommodation and flights unrelated to this work. PJMO reports personal fees from consultancy, grants from MRC, EU, NIHR Biomedical Research Centres, MRC/GSK, Wellcome Trust, NIHR (HPRU) and NIHR Senior Investigator Award. Personal fees from European Respiratory Society, grants from MRC Global Challenge Research fund, other from Nestle Discussion Forum (unpaid), Pfizer antivirals advisory board (unpaid) outside of the submitted work and the role of President of the British Society for Immunology was an unpaid appointment but PJMO's travel and accommodation at some meetings is provided by the Society. MGS reports grants from the National Institute for Health Research (NIHR), Medical Research Council. NIHR Health Protection Research Unit (HPRU) in Emerging and Zoonotic Infections at University of Liverpool in partnership with Public Health England (PHE), in collaboration with Liverpool School of Tropical Medicine and the University of Oxford. All other authors have no interests to declare.

## Appendix Supplementary materials

Image, application 1

Image, application 2

Image, application 3

Image, application 4

Image, application 5

Image, application 6

Image, application 7

Image, application 8

Image, application 9

Image, application 10

Image, application 11

Image, application 12

## Footnotes

Supplementary material associated with this article can be found in the online version at doi:10.1016/j.lanepe.2021.100186.
