# Supplementary material for: Long Covid in adults discharged from UK hospitals after Covid-19: A prospective, multicentre cohort study using the ISARIC WHO Clinical Characterisation Protocol
Source: Lancet Reg Health Eur. 2021 Aug 6;8:100186. doi: 10.1016/j.lanepe.2021.100186 (PMC8343377; doi:10.1016/j.lanepe.2021.100186)
Supplement: Supplementary file 1 [file mmc1.docx]

**Supplementary appendix 1**

**Supplementary table 1 –** Comparison between respondents and those who did not respond

|  |  | No response to questionnaire | Responded to questionnaire | p-value |
| --- | --- | --- | --- | --- |
| Total N (%) |  | 497 (60$\cdot$3) | 327 (39$\cdot$7) |  |
| Age | Median (IQR) | 57$\cdot$7 (48$\cdot$1 to 69$\cdot$6) | 59$\cdot$7 (51.7 to 67$\cdot$7) | 0$\cdot$173 |
|  | <50 | 138 (27$\cdot$8) | 70 (21$\cdot$4) |  |
|  | 50-69 | 240 (48$\cdot$3) | 195 (59$\cdot$6) |  |
|  | 70-79 | 69 (13$\cdot$9) | 40 (12$\cdot$2) |  |
|  | 80+ | 50 (10$\cdot$1) | 22 (6$\cdot$7) |  |
| Sex at Birth | Male | 302 (60$\cdot$8) | 192 (58$\cdot$7) | 0$\cdot$559 |
|  | Female | 193 (38$\cdot$8) | 135 (41$\cdot$3) |  |
|  | (Missing) | 2 (0$\cdot$4) | 0 (0$\cdot$0) |  |
| Ethnicity | White | 331 (66$\cdot$6) | 265 (81$\cdot$0) | <0$\cdot$001 |
|  | South Asian | 37 (7$\cdot$4) | 8 (2$\cdot$4) |  |
|  | East Asian | 16 (3$\cdot$2) | 4 (1$\cdot$2) |  |
|  | Black | 40 (8$\cdot$0) | 15 (4$\cdot$6) |  |
|  | Other Ethnic Minority | 44 (8$\cdot$9) | 21 (6$\cdot$4) |  |
|  | (Missing) | 29 (5$\cdot$8) | 14 (4$\cdot$3) |  |
| Smoking | Never Smoked | 274 (55$\cdot$1) | 176 (53$\cdot$8) | 0$\cdot$018 |
|  | Current Smoker | 31 (6$\cdot$2) | 7 (2$\cdot$1) |  |
|  | Former Smoker | 123 (24$\cdot$7) | 92 (28$\cdot$1) |  |
|  | (Missing) | 69 (13$\cdot$9) | 52 (15$\cdot$9) |  |
| Diabetes | No | 388 (80$\cdot$8) | 254 (80$\cdot$4) | 0$\cdot$947 |
|  | Yes | 92 (19$\cdot$2) | 62 (19$\cdot$6) |  |
| Obesity (as defined by clinical staff) | No | 407 (81$\cdot$9) | 254 (77$\cdot$7) | 0$\cdot$069 |
|  | Yes | 61 (12$\cdot$3) | 56 (17$\cdot$1) |  |
|  | (Missing) | 29 (5$\cdot$8) | 17 (5$\cdot$2) |  |
| Chronic cardiac disease | No | 396 (79$\cdot$7) | 272 (83$\cdot$2) | 0$\cdot$213 |
|  | Yes | 84 (16$\cdot$9) | 44 (13$\cdot$5) |  |
|  | (Missing) | 17 (3$\cdot$4) | 11 (3$\cdot$4) |  |
| Chronic pulmonary disease (not asthma) | No | 428 (86$\cdot$1) | 290 (88$\cdot$7) | 0$\cdot$130 |
|  | Yes | 52 (10$\cdot$5) | 23 (7$\cdot$0) |  |
|  | (Missing) | 17 (3$\cdot$4) | 14 (4$\cdot$3) |  |
| Asthma (physician diagnosed) | No | 399 (80$\cdot$3) | 252 (77$\cdot$1) | 0$\cdot$255 |
|  | Yes | 82 (16$\cdot$5) | 65 (19$\cdot$9) |  |
|  | (Missing) | 16 (3$\cdot$2) | 10 (3$\cdot$1) |  |
| Chronic kidney disease | No | 442 (88$\cdot$9) | 298 (91$\cdot$1) | 0$\cdot$181 |
|  | Yes | 41 (8$\cdot$2) | 18 (5$\cdot$5) |  |
|  | (Missing) | 14 (2$\cdot$8) | 11 (3$\cdot$4) |  |
| Malignant neoplasm | No | 457 (92$\cdot$0) | 306 (93$\cdot$6) | 0$\cdot$192 |
|  | Yes | 26 (5$\cdot$2) | 10 (3$\cdot$1) |  |
|  | (Missing) | 14 (2$\cdot$8) | 11 (3$\cdot$4) |  |
| Rheumatologic disorder | No | 445 (89$\cdot$5) | 290 (88$\cdot$7) | 0$\cdot$719 |
|  | Yes | 32 (6$\cdot$4) | 24 (7$\cdot$3) |  |
|  | (Missing) | 20 (4$\cdot$0) | 13 (4$\cdot$0) |  |
| Critical care admission | Ward level care only | 363 (73$\cdot$0) | 197 (60$\cdot$2) | <0$\cdot$001 |
|  | Admitted to Critical Care | 133 (26$\cdot$8) | 130 (39$\cdot$8) |  |
|  | (Missing) | 1 (0$\cdot$2) | 0 (0$\cdot$0) |  |
| Length of stay (days) | Median (IQR) | 8.0 (5.0 to 20$\cdot$0) | 9.0 (5.0 to 20$\cdot$0) | 0$\cdot$486 |
