# Supplementary figures and images for: Long Covid in adults discharged from UK hospitals after Covid-19: A prospective, multicentre cohort study using the ISARIC WHO Clinical Characterisation Protocol
Source: Lancet Reg Health Eur. 2021 Aug 6;8:100186. doi: 10.1016/j.lanepe.2021.100186 (PMC8343377; doi:10.1016/j.lanepe.2021.100186)

**Supplementary Figure 1 –** Fatigue rating on Visual Analogue Scale (VAS) by sex.


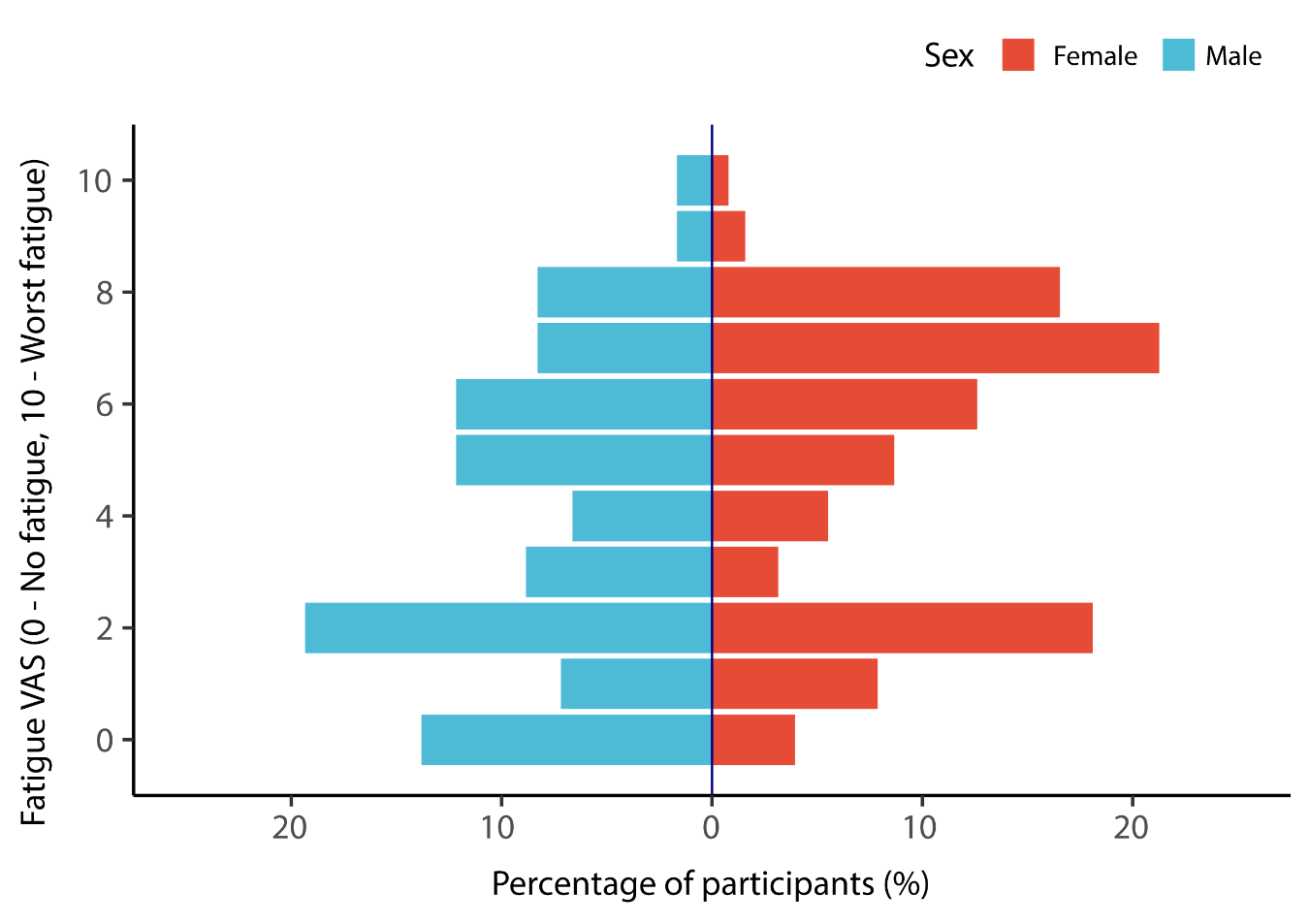

Supplement: Supplementary file 2 [file mmc2.docx]
