# Supplementary material for: Long Covid in adults discharged from UK hospitals after Covid-19: A prospective, multicentre cohort study using the ISARIC WHO Clinical Characterisation Protocol
Source: Lancet Reg Health Eur. 2021 Aug 6;8:100186. doi: 10.1016/j.lanepe.2021.100186 (PMC8343377; doi:10.1016/j.lanepe.2021.100186)
Supplement: Supplementary file 3 [file mmc3.docx]

**Supplementary table 2 –** Long-term outcomes by sex

|  |  | Male | Female | p-value |
| --- | --- | --- | --- | --- |
| Total N (%) |  | 192 (58.7) | 135 (41$\cdot$3) |  |
| Self-reported overall recovery | Feels fully recovered | 56 (29.2) | 27 (20$\cdot$0) | 0$\cdot$117 |
|  | Does not feel fully recovered | 103 (53.6) | 76 (56$\cdot$3) |  |
|  | Not sure | 31 (16.1) | 30 (22$\cdot$2) |  |
|  | (Missing) | 2 (1.0) | 2 (1$\cdot$5) |  |
| New or persistent symptoms | No new or persistent symptoms | 17 (8.9) | 5 (3$\cdot$7) | 0$\cdot$108 |
|  | New or persistent symptoms | 175 (91.1) | 130 (96$\cdot$3) |  |
| Change in breathlessness after COVID-19 (MRC Dyspnoea) | No change | 88 (45.8) | 39 (28$\cdot$9) | 0$\cdot$015 |
|  | Less breathless | 7 (3.6) | 4 (3$\cdot$0) |  |
|  | More breathless | 80 (41.7) | 73 (54$\cdot$1) |  |
|  | (Missing) | 17 (8.9) | 19 (14$\cdot$1) |  |
| Fatigue level (0 to 10 VAS) | Median (IQR) | 4.0 (2.0 to 6.0) | 6$\cdot$0 (2$\cdot$0 to 7$\cdot$0) | <0$\cdot$001 |
| EQ5D-5L change in overall summary index | Median (IQR) | -0.0 (-0.2 to 0.0) | -0$\cdot$1 (-0$\cdot$3 to 0$\cdot$0) | <0$\cdot$001 |
| Washington Group Short Set | No change in disability | 150 (78.1) | 91 (6$\cdot$4) | 0$\cdot$059 |
|  | New or worse disability in at least one domain | 39 (20.3) | 40 (29$\cdot$6) |  |
|  | (Missing) | 3 (1.6) | 4 (3$\cdot$0) |  |

HFNC – High flow nasal cannulae, NIV – Noninvasive ventilation, MRC – Medical Research Council, IQR – Interquartile range, presented as 25^th^ to 75^th^ centiles. Numbers are presented as N (%), unless otherwise denoted as a continuous variable.
