# Supplementary material for: Long Covid in adults discharged from UK hospitals after Covid-19: A prospective, multicentre cohort study using the ISARIC WHO Clinical Characterisation Protocol
Source: Lancet Reg Health Eur. 2021 Aug 6;8:100186. doi: 10.1016/j.lanepe.2021.100186 (PMC8343377; doi:10.1016/j.lanepe.2021.100186)
Supplement: Supplementary file 4 [file mmc4.docx]

**Supplementary table 3 –**Overall new or worse disability by Washington Group disability domains before onset of Covid-19 compared with disability at time of follow-up.

| Washington Group Domain |  | Total participants (%) |
| --- | --- | --- |
| Total N (%) |  | 327 (100$\cdot$0) |
| Vision | No change | 256 (78$\cdot$3) |
|  | New mild disability | 57 (17$\cdot$4) |
|  | New moderate disability | 3 (0$\cdot$9) |
|  | New severe disability | 0 (0$\cdot$0) |
|  | (Missing) | 11 (3$\cdot$4) |
| Hearing | No change | 286 (87$\cdot$5) |
|  | New mild disability | 28 (8$\cdot$6) |
|  | New moderate disability | 4 (1$\cdot$2) |
|  | New severe disability | 0 (0$\cdot$0) |
|  | (Missing) | 9 (2$\cdot$8) |
| Walking and mobility | No change | 181 (55$\cdot$4) |
|  | New mild disability | 109 (33$\cdot$3) |
|  | New moderate disability | 21 (6$\cdot$4) |
|  | New severe disability | 1 (0$\cdot$3) |
|  | (Missing) | 15 (4$\cdot$6) |
| Memory and concentration | No change | 182 (55$\cdot$7) |
|  | New mild disability | 98 (30$\cdot$0) |
|  | New moderate disability | 32 (9$\cdot$8) |
|  | New severe disability | 0 (0$\cdot$0) |
|  | (Missing) | 15 (4$\cdot$6) |
| Washing and self-care | No change | 268 (82$\cdot$0) |
|  | New mild disability | 45 (13$\cdot$8) |
|  | New moderate disability | 4 (1$\cdot$2) |
|  | New severe disability | 0 (0$\cdot$0) |
|  | (Missing) | 10 (3$\cdot$1) |
| Communicating | No change | 265 (81$\cdot$0) |
|  | New mild disability | 50 (15$\cdot$3) |
|  | New moderate disability | 3 (0$\cdot$9) |
|  | New severe disability | 0 (0$\cdot$0) |
|  | (Missing) | 9 (2$\cdot$8) |

Numbers are N (%).
