# Supplementary material for: Long Covid in adults discharged from UK hospitals after Covid-19: A prospective, multicentre cohort study using the ISARIC WHO Clinical Characterisation Protocol
Source: Lancet Reg Health Eur. 2021 Aug 6;8:100186. doi: 10.1016/j.lanepe.2021.100186 (PMC8343377; doi:10.1016/j.lanepe.2021.100186)
Supplement: Supplementary file 5 [file mmc5.docx]

**Supplementary table 4 –** New or worse disability across Washington Group disability domains, before onset of Covid-19 compared with disability at time of follow-up stratified by sex.

| Washington Group Domain |  | Male | Female | p-value |
| --- | --- | --- | --- | --- |
| Total N (%) |  | 192 (58$\cdot$7) | 135 (41$\cdot$3) |  |
| Vision | No change | 160 (83$\cdot$3) | 96 (71$\cdot$1) | 0$\cdot$009 |
|  | New mild disability | 23 (12$\cdot$0) | 34 (25$\cdot$2) |  |
|  | New moderate disability | 2 (1$\cdot$0) | 1 (0$\cdot$7) |  |
|  | New severe disability | 0 (0$\cdot$0) | 0 (0$\cdot$0) |  |
|  | (Missing) | 7 (3$\cdot$6) | 4 (3$\cdot$0) |  |
| Hearing | No change | 168 (87$\cdot$5) | 118 (87$\cdot$4) | 0$\cdot$246 |
|  | New mild disability | 17 (8$\cdot$9) | 11 (8$\cdot$1) |  |
|  | New moderate disability | 4 (2$\cdot$1) | 0 (0$\cdot$0) |  |
|  | New severe disability | 0 (0$\cdot$0) | 0 (0$\cdot$0) |  |
|  | (Missing) | 3 (1$\cdot$6) | 6 (4$\cdot$4) |  |
| Walking and mobility | No change | 120 (62$\cdot$5) | 61 (45$\cdot$2) | 0$\cdot$040 |
|  | New mild disability | 55 (28$\cdot$6) | 54 (40$\cdot$0) |  |
|  | New moderate disability | 11 (5$\cdot$7) | 10 (7$\cdot$4) |  |
|  | New severe disability | 1 (0$\cdot$5) | 0 (0$\cdot$0) |  |
|  | (Missing) | 5 (2$\cdot$6) | 10 (7$\cdot$4) |  |
| Memory and concentration | No change | 119 (62$\cdot$0) | 63 (46$\cdot$7) | 0$\cdot$023 |
|  | New mild disability | 52 (27$\cdot$1) | 46 (34$\cdot$1) |  |
|  | New moderate disability | 14 (7$\cdot$3) | 18 (13$\cdot$3) |  |
|  | New severe disability | 0 (0$\cdot$0) | 0 (0$\cdot$0) |  |
|  | (Missing) | 7 (3$\cdot$6) | 8 (5$\cdot$9) |  |
| Washing and self-care | No change | 161 (83$\cdot$9) | 107 (79$\cdot$3) | 0$\cdot$651 |
|  | New mild disability | 24 (12$\cdot$5) | 21 (15$\cdot$6) |  |
|  | New moderate disability | 2 (1$\cdot$0) | 2 (1$\cdot$5) |  |
|  | New severe disability | 0 (0$\cdot$0) | 0 (0$\cdot$0) |  |
|  | (Missing) | 5 (2$\cdot$6) | 5 (3$\cdot$7) |  |
| Communicating | No change | 159 (82$\cdot$8) | 106 (78$\cdot$5) | 0$\cdot$552 |
|  | New mild disability | 26 (13$\cdot$5) | 24 (17$\cdot$8) |  |
|  | New moderate disability | 2 (1$\cdot$0) | 1 (0$\cdot$7) |  |
|  | New severe disability | 0 (0$\cdot$0) | 0 (0$\cdot$0) |  |
|  | (Missing) | 5 (2$\cdot$6) | 4 (3$\cdot$0) |  |

Numbers are N (%).
