# Supplementary material for: Long Covid in adults discharged from UK hospitals after Covid-19: A prospective, multicentre cohort study using the ISARIC WHO Clinical Characterisation Protocol
Source: Lancet Reg Health Eur. 2021 Aug 6;8:100186. doi: 10.1016/j.lanepe.2021.100186 (PMC8343377; doi:10.1016/j.lanepe.2021.100186)
Supplement: Supplementary file 6 [file mmc6.docx]

**Supplementary table 5 –** Overall changes by EQ5D-5L dimension before Covid-19 onset and at time of follow-up.

| Dimension | Change in EQ5D-5L | Total number of participants (%) |
| --- | --- | --- |
| Mobility | No change | 199 (64$\cdot$0) |
|  | Improve | 3 (1$\cdot$0) |
|  | Worsen | 109 (35$\cdot$0) |
|  | Total | 311 (100$\cdot$0) |
| Self-care | No change | 256 (82$\cdot$3) |
|  | Improve | 1 (0$\cdot$3) |
|  | Worsen | 54 (17$\cdot$4) |
|  | Total | 311 (100$\cdot$0) |
| Usual activities | No change | 185 (59$\cdot$5) |
|  | Improve | 5 (1$\cdot$6) |
|  | Worsen | 121 (38$\cdot$9) |
|  | Total | 311 (100$\cdot$0) |
| Pain/discomfort | No change | 184 (59$\cdot$2) |
|  | Improve | 10 (3$\cdot$2) |
|  | Worsen | 117 (37$\cdot$6) |
|  | Total | 311 (100$\cdot$0) |
| Anxiety/depression | No change | 181 (58$\cdot$2) |
|  | Improve | 13 (4$\cdot$2) |
|  | Worsen | 117 (37$\cdot$6) |
|  | Total | 311 (100$\cdot$0) |

Numbers are N (%).
