# Supplementary material for: Long Covid in adults discharged from UK hospitals after Covid-19: A prospective, multicentre cohort study using the ISARIC WHO Clinical Characterisation Protocol
Source: Lancet Reg Health Eur. 2021 Aug 6;8:100186. doi: 10.1016/j.lanepe.2021.100186 (PMC8343377; doi:10.1016/j.lanepe.2021.100186)
Supplement: Supplementary file 7 [file mmc7.docx]

**Supplementary table 6 –** Overall changes by EQ5D-5L dimension and sex before Covid-19 onset and at time of follow-up using the Paretian change approach.

| Dimension | Change in EQ5D-5L | Male (%) | Female (%) |
| --- | --- | --- | --- |
| Mobility | No change | 126 (68$\cdot$1) | 73 (57$\cdot$9) |
|  | Improve | 2 (1$\cdot$1) | 1 (0$\cdot$8) |
|  | Worsen | 57 (30$\cdot$8) | 52 (41$\cdot$3) |
|  | Total | 185 (100$\cdot$0) | 126 (100$\cdot$0) |
| Self-care | No change | 156 (84$\cdot$3) | 100 (79$\cdot$4) |
|  | Improve | 1 (0$\cdot$5) | 0 (0$\cdot$0) |
|  | Worsen | 28 (15$\cdot$1) | 26 (20$\cdot$6) |
|  | Total | 185 (100$\cdot$0) | 126 (100$\cdot$0) |
| Usual activities | No change | 120 (64$\cdot$9) | 65 (51$\cdot$6) |
|  | Improve | 3 (1$\cdot$6) | 2 (1$\cdot$6) |
|  | Worsen | 62 (33$\cdot$5) | 59 (46$\cdot$8) |
|  | Total | 185 (100$\cdot$0) | 126 (100$\cdot$0) |
| Pain/discomfort | No change | 121 (65$\cdot$4) | 63 (50$\cdot$0) |
|  | Improve | 6 (3$\cdot$2) | 4 (3$\cdot$2) |
|  | Worsen | 58 (31$\cdot$4) | 59 (46$\cdot$8) |
|  | Total | 185 (100$\cdot$0) | 126 (100$\cdot$0) |
| Anxiety/depression | No change | 126 (68$\cdot$1) | 55 (43$\cdot$7) |
|  | Improve | 8 (4$\cdot$3) | 5 (4$\cdot$0) |
|  | Worsen | 51 (27$\cdot$6) | 66 (52$\cdot$4) |
|  | Total | 185 (100$\cdot$0) | 126 (100$\cdot$0) |

Numbers are N (%).
