# Supplementary material for: Long Covid in adults discharged from UK hospitals after Covid-19: A prospective, multicentre cohort study using the ISARIC WHO Clinical Characterisation Protocol
Source: Lancet Reg Health Eur. 2021 Aug 6;8:100186. doi: 10.1016/j.lanepe.2021.100186 (PMC8343377; doi:10.1016/j.lanepe.2021.100186)
Supplement: Supplementary file 8 [file mmc8.docx]

**Supplementary table 7 –** Proportion of participants experiencing new or persistent symptoms at time of follow-up.

| Symptom | Number of participants experiencing symptom (%) | Participant denominator |
| --- | --- | --- |
| Fatigue | 255 (82$\cdot$8) | 308 |
| Shortness of breath | 175 (53$\cdot$5) | 327 |
| Problems sleeping | 151 (46$\cdot$2) | 327 |
| Headache | 129 (39$\cdot$4) | 327 |
| Limb weakness | 123 (37$\cdot$6) | 327 |
| Joint pain or swelling | 121 (37$\cdot$0) | 327 |
| Persistent muscle pain | 121 (37$\cdot$0) | 327 |
| Dizziness/light headedness | 117 (35$\cdot$8) | 327 |
| Problems with balance | 99 (30$\cdot$3) | 327 |
| Swollen ankle | 80 (24$\cdot$5) | 327 |
| Palpitations | 76 (23$\cdot$2) | 327 |
| Constipation | 59 (18$\cdot$0) | 327 |
| Problems seeing | 59 (18$\cdot$0) | 327 |
| Diarrhoea | 58 (17$\cdot$7) | 327 |
| Stomach pain | 58 (17$\cdot$7) | 327 |
| Chest pains | 50 (15$\cdot$3) | 327 |
| Persistent cough | 50 (15$\cdot$3) | 327 |
| Erectile dysfunction | 45 (23$\cdot$4) | 192 |
| Pain on breathing | 43 (13$\cdot$1) | 327 |
| Loss of smell | 42 (12$\cdot$8) | 327 |
| Other | 42 (12$\cdot$8) | 327 |
| Persistent fevers | 36 (11$\cdot$0) | 327 |
| Loss of taste | 35 (10$\cdot$7) | 327 |
| Nausea/vomiting | 34 (10$\cdot$4) | 327 |
| Loss of appetite | 33 (10$\cdot$1) | 327 |
| Problems swallowing | 28 (8$\cdot$6) | 327 |
| Skin rash | 27 (8$\cdot$3) | 327 |
| Weight loss | 24 (7$\cdot$3) | 327 |
| Problems passing urine | 23 (7$\cdot$0) | 327 |
| Hemiplegia/paraesthesiae | 20 (6$\cdot$1) | 327 |
| Toe lesions | 13 (4$\cdot$0) | 327 |
