# Supplementary material for: Long Covid in adults discharged from UK hospitals after Covid-19: A prospective, multicentre cohort study using the ISARIC WHO Clinical Characterisation Protocol
Source: Lancet Reg Health Eur. 2021 Aug 6;8:100186. doi: 10.1016/j.lanepe.2021.100186 (PMC8343377; doi:10.1016/j.lanepe.2021.100186)
Supplement: Supplementary file 9 [file mmc9.docx]

**Supplementary table 8 –** Differences by sex for comorbidities and participant demographics

| label | levels | Male | Female | Total | p |
| --- | --- | --- | --- | --- | --- |
| Total N (%) |  | 192 (58.7) | 135 (41.3) | 327 |  |
| Age | Median (IQR) | 60.2 (53.8 to 68.7) | 59.3 (49.5 to 65.6) | 59.7 (51.7 to 67.7) | 0.060 |
|  | Under 50 | 34 (48.6) | 36 (51.4) | 70 (100) |  |
|  | 50 to 69 | 114 (58.5) | 81 (41.5) | 195 (100) |  |
|  | Over 70 | 44 (71.0) | 18 (29.0) | 62 (100) |  |
| Ethnicity | White | 151 (57.0) | 114 (43.0) | 265 (100) | 0.891 |
|  | South Asian | 5 (62.5) | 3 (37.5) | 8 (100) |  |
|  | East Asian | 3 (75.0) | 1 (25.0) | 4 (100) |  |
|  | Black | 10 (66.7) | 5 (33.3) | 15 (100) |  |
|  | Other Ethnic Minority | 12 (57.1) | 9 (42.9) | 21 (100) |  |
|  | (Missing) | 11 (78.6) | 3 (21.4) | 14 (100) |  |
| Smoking | Never Smoked | 98 (55.7) | 78 (44.3) | 176 (100) | 0.167 |
|  | Current Smoker | 3 (42.9) | 4 (57.1) | 7 (100) |  |
|  | Former Smoker | 61 (66.3) | 31 (33.7) | 92 (100) |  |
|  | (Missing) | 30 (57.7) | 22 (42.3) | 52 (100) |  |
| Diabetes | No | 146 (57.5) | 108 (42.5) | 254 (100) | 0.864 |
|  | Yes | 37 (59.7) | 25 (40.3) | 62 (100) |  |
| Obesity (as defined by clinical staff) | No | 147 (57.9) | 107 (42.1) | 254 (100) | 0.845 |
|  | Yes | 31 (55.4) | 25 (44.6) | 56 (100) |  |
|  | (Missing) | 14 (82.4) | 3 (17.6) | 17 (100) |  |
| Chronic cardiac disease | No | 150 (55.1) | 122 (44.9) | 272 (100) | 0.021 |
|  | Yes | 33 (75.0) | 11 (25.0) | 44 (100) |  |
|  | (Missing) | 9 (81.8) | 2 (18.2) | 11 (100) |  |
| Chronic pulmonary disease (not asthma) | No | 164 (56.6) | 126 (43.4) | 290 (100) | 0.319 |
|  | Yes | 16 (69.6) | 7 (30.4) | 23 (100) |  |
|  | (Missing) | 12 (85.7) | 2 (14.3) | 14 (100) |  |
| Asthma (physician diagnosed) | No | 153 (60.7) | 99 (39.3) | 252 (100) | 0.048 |
|  | Yes | 30 (46.2) | 35 (53.8) | 65 (100) |  |
|  | (Missing) | 9 (90.0) | 1 (10.0) | 10 (100) |  |
| Chronic kidney disease | No | 169 (56.7) | 129 (43.3) | 298 (100) | 0.130 |
|  | Yes | 14 (77.8) | 4 (22.2) | 18 (100) |  |
|  | (Missing) | 9 (81.8) | 2 (18.2) | 11 (100) |  |
| Malignant neoplasm | No | 177 (57.8) | 129 (42.2) | 306 (100) | 1.000 |
|  | Yes | 6 (60.0) | 4 (40.0) | 10 (100) |  |
|  | (Missing) | 9 (81.8) | 2 (18.2) | 11 (100) |  |
| Rheumatologic disorder | No | 172 (59.3) | 118 (40.7) | 290 (100) | 0.142 |
|  | Yes | 10 (41.7) | 14 (58.3) | 24 (100) |  |
|  | (Missing) | 10 (76.9) | 3 (23.1) | 13 (100) |  |
| ISARIC-4C Mortality Score (predicted severity) | Median (IQR) | 8.0 (5.0 to 10.0) | 6.0 (3.0 to 7.0) | 7.0 (4.0 to 9.0) | <0.001 |
| Severity | Scale 3 (did not require supplemental oxygen) | 30 (44.1) | 38 (55.9) | 68 (100) | 0.007 |
|  | Scale 4 (required supplemental oxygen) | 66 (55.9) | 52 (44.1) | 118 (100) |  |
|  | Scale 5 (required HFNC or NIV) | 31 (63.3) | 18 (36.7) | 49 (100) |  |
|  | Scale 6 (required invasive mechanical ventilation) | 65 (70.7) | 27 (29.3) | 92 (100) |  |
| Critical care admission | Ward level care only | 101 (51.3) | 96 (48.7) | 197 (100) | 0.001 |
|  | Admitted to Critical Care | 91 (70.0) | 39 (30.0) | 130 (100) |  |
| Length of stay (days) | Median (IQR) | 11.0 (6.0 to 22.2) | 8.0 (5.0 to 13.0) | 9.0 (5.0 to 20.0) | 0.002 |
| Time from symptoms to completing survey (days) | Median (IQR) | 220.5 (188.0 to 268.2) | 224.0 (190.0 to 268.0) | 222.0 (189.0 to 268.5) | 0.882 |
| Time from discharge to completing survey (days) | Median (IQR) | 194.5 (161.0 to 249.5) | 204.0 (176.5 to 248.5) | 200.0 (171.0 to 249.0) | 0.277 |

HFNC – High flow nasal cannulae, NIV – Noninvasive ventilation, IQR – Interquartile range, presented as 25^th^ to 75^th^ centiles. Numbers are presented as N (%), unless otherwise denoted as a continuous variable.
