# Supplementary material for: Long Covid in adults discharged from UK hospitals after Covid-19: A prospective, multicentre cohort study using the ISARIC WHO Clinical Characterisation Protocol
Source: Lancet Reg Health Eur. 2021 Aug 6;8:100186. doi: 10.1016/j.lanepe.2021.100186 (PMC8343377; doi:10.1016/j.lanepe.2021.100186)
Supplement: Supplementary file 10 [file mmc10.docx]

**Supplementary table 9 –** Differences in severity of in-hospital disease by sex

SD – Standard Deviation

|  |  | Male | Female | p |
| --- | --- | --- | --- | --- |
| ISARIC-4C Mortality Score (predicted severity) | Mean (SD) | 7.7 (3.4) | 5.5 (2.7) | <0.001 |
| Length of stay (days) | Mean (SD) | 18.1 (18.8) | 12.7 (16.3) | 0.009 |
| Any invasive mechanical ventilation | No | 127 (66.1) | 108 (80.0) | 0.009 |
|  | Yes | 65 (33.9) | 27 (20.0) |  |
| Critical care admission | Ward level care only | 101 (52.6) | 96 (71.1) | 0.001 |
|  | Admitted to Critical Care | 91 (47.4) | 39 (28.9) |  |
