# Supplementary material for: Long Covid in adults discharged from UK hospitals after Covid-19: A prospective, multicentre cohort study using the ISARIC WHO Clinical Characterisation Protocol
Source: Lancet Reg Health Eur. 2021 Aug 6;8:100186. doi: 10.1016/j.lanepe.2021.100186 (PMC8343377; doi:10.1016/j.lanepe.2021.100186)
Supplement: Supplementary file 11 [file mmc11.docx]

**Supplementary table 10 –** Outcomes in those testing positive for SARS-CoV-2

| label | levels | Scale 3 (did not require supplemental oxygen) | Scale 4 (required supplemental oxygen) | Scale 5 (required HFNC or NIV) | Scale 6 (required invasive mechanical ventilation) | p |
| --- | --- | --- | --- | --- | --- | --- |
| Total N (%) |  | 67 (20.7) | 117 (36.2) | 49 (15.2) | 90 (27.9) |  |
| Do you feel fully recovered from COVID-19? | Feels fully recovered | 21 (31.3) | 33 (28.2) | 11 (22.4) | 16 (17.8) | 0.003 |
|  | Does not feel fully recovered | 33 (49.3) | 53 (45.3) | 27 (55.1) | 66 (73.3) |  |
|  | Not sure | 12 (17.9) | 29 (24.8) | 11 (22.4) | 7 (7.8) |  |
|  | (Missing) | 1 (1.5) | 2 (1.7) | 0 (0.0) | 1 (1.1) |  |
| Persistent Symptoms | No persistent symptoms | 3 (4.5) | 9 (7.7) | 6 (12.2) | 4 (4.4) | 0.285 |
|  | Persistent symptoms | 64 (95.5) | 108 (92.3) | 43 (87.8) | 86 (95.6) |  |
| Change in breathlessness after COVID-19 | No change | 25 (37.3) | 54 (46.2) | 20 (40.8) | 25 (27.8) | 0.046 |
|  | Less breathless | 2 (3.0) | 5 (4.3) | 2 (4.1) | 2 (2.2) |  |
|  | More breathless | 31 (46.3) | 41 (35.0) | 24 (49.0) | 56 (62.2) |  |
|  | (Missing) | 9 (13.4) | 17 (14.5) | 3 (6.1) | 7 (7.8) |  |
| Fatigue level | Median (IQR) | 6.0 (2.0 to 7.0) | 4.0 (2.0 to 7.0) | 5.0 (2.0 to 7.0) | 4.0 (2.0 to 7.0) | 0.486 |
| EQ5D-5L Overall change in health state | Median (IQR) | -0.1 (-0.2 to 0.0) | -0.0 (-0.2 to 0.0) | -0.1 (-0.2 to 0.0) | -0.1 (-0.3 to 0.0) | 0.007 |
| Washington Group Short Set | No change in disability | 51 (76.1) | 87 (74.4) | 35 (71.4) | 64 (71.1) | 0.887 |
|  | New disability in at least one domain | 15 (22.4) | 27 (23.1) | 13 (26.5) | 24 (26.7) |  |
|  | (Missing) | 1 (1.5) | 3 (2.6) | 1 (2.0) | 2 (2.2) |  |

 HFNC – High flow nasal cannulae, NIV – Noninvasive ventilation, MRC – Medical Research Council, IQR – Interquartile range, presented as 25^th^ to 75^th^ centiles. Numbers are presented as N (%), unless otherwise denoted as a continuous variable.
