# Supplementary material for: Long Covid in adults discharged from UK hospitals after Covid-19: A prospective, multicentre cohort study using the ISARIC WHO Clinical Characterisation Protocol
Source: Lancet Reg Health Eur. 2021 Aug 6;8:100186. doi: 10.1016/j.lanepe.2021.100186 (PMC8343377; doi:10.1016/j.lanepe.2021.100186)
Supplement: Supplementary file 12 [file mmc12.docx]

**Supplementary table 11 –** Multilevel regression models including only those testing positive for SARS-CoV-2.

| Explanatory variable |  | Overall recovery: OR (multilevel) | Persistent symptoms: OR (multilevel) | Change in MRC Dyspnoea: OR (multilevel) | Fatigue level: Coefficient (multilevel) | EQ5D-5L health state change: Coefficient (multilevel) | Washington Short Set Disability: OR (multilevel) |
| --- | --- | --- | --- | --- | --- | --- | --- |
| Sex at Birth:Age | Male \| Under 50 | - | - | - | - | - | - |
|  | Male \| 50 to 69 | 1.61 (0.68-3.82, p=0.280) | 0.80 (0.20-3.22, p=0.756) | 2.35 (0.95-5.83, p=0.066) | 0.43 (-0.58 to 1.44, p=0.204) | -0.04 (-0.11 to 0.02, p=0.103) | 1.75 (0.53-5.72, p=0.357) |
|  | Male \| Over 70 | 1.47 (0.52-4.19, p=0.471) | 0.73 (0.14-3.78, p=0.710) | 2.43 (0.79-7.47, p=0.122) | 0.35 (-0.89 to 1.58, p=0.292) | -0.04 (-0.12 to 0.04, p=0.175) | 2.17 (0.57-8.30, p=0.260) |
|  | Female \| Under 50 | 5.05 (1.63-15.67, p=0.005) | 2.75 (0.26-29.00, p=0.399) | 7.05 (2.22-22.43, p=0.001) | 2.07 (0.82 to 3.33, p=0.001) | -0.19 (-0.27 to -0.11, p<0.001) | 4.23 (1.12-15.97, p=0.034) |
|  | Female \| 50 to 69 | 1.75 (0.71-4.32, p=0.227) | 2.10 (0.39-11.38, p=0.388) | 6.17 (2.28-16.74, p<0.001) | 1.20 (0.15 to 2.25, p=0.013) | -0.10 (-0.17 to -0.03, p=0.003) | 2.70 (0.81-9.07, p=0.107) |
|  | Female \| Over 70 | 0.41 (0.10-1.68, p=0.215) | 1.22 (0.11-13.95, p=0.873) | 0.64 (0.13-3.22, p=0.593) | 0.29 (-1.34 to 1.92, p=0.364) | -0.06 (-0.17 to 0.04, p=0.113) | 1.90 (0.36-9.92, p=0.447) |
| Any comorbidity | No comorbidities | - | - | - | - | - | - |
|  | One or more comorbidities | 0.86 (0.50-1.45, p=0.565) | 2.28 (0.92-5.65, p=0.076) | 0.76 (0.43-1.34, p=0.345) | 0.93 (0.32 to 1.54, p=0.001) | -0.02 (-0.06 to 0.02, p=0.149) | 3.03 (1.61-5.71, p=0.001) |
| Severity | Scale 3 (did not require supplemental oxygen) | - | - | - | - | - | - |
|  | Scale 4 (required supplemental oxygen) | 0.93 (0.47-1.87, p=0.845) | 0.61 (0.15-2.44, p=0.486) | 0.54 (0.25-1.13, p=0.102) | -0.22 (-1.04 to 0.59, p=0.296) | 0.04 (-0.01 to 0.10, p=0.070) | 1.08 (0.49-2.35, p=0.851) |
|  | Scale 5 (required HFNC or NIV) | 1.50 (0.61-3.67, p=0.375) | 0.33 (0.07-1.49, p=0.150) | 0.90 (0.36-2.23, p=0.813) | -0.17 (-1.21 to 0.86, p=0.373) | 0.01 (-0.06 to 0.08, p=0.369) | 1.26 (0.47-3.38, p=0.641) |
|  | Scale 6 (required invasive mechanical ventilation) | 3.78 (1.64-8.71, p=0.002) | 1.19 (0.24-5.98, p=0.832) | 1.99 (0.85-4.64, p=0.110) | -0.17 (-1.10 to 0.76, p=0.361) | -0.04 (-0.10 to 0.02, p=0.088) | 1.49 (0.63-3.54, p=0.367) |
